# Influence of Polycation Composition on Electrochemical Film Formation

**DOI:** 10.3390/polym10040429

**Published:** 2018-04-12

**Authors:** Sabine Schneider, Corinna Janssen, Elisabeth Klindtworth, Olga Mergel, Martin Möller, Felix Plamper

**Affiliations:** 1 Institute of Physical Chemistry, RWTH Aachen University, Landoltweg 2, 52056 Aachen, Germany; sabine.schneider@pc.rwth-aachen.de (S.S.); corinna.janssen@rwth-aachen.de (C.J.); elisabeth.klindtworth@rwth-aachen.de (E.K.); o.mergel@umcg.nl (O.M.); 2 Department of Biomedical Engineering-FB40, University of Groningen, University Medical Center Groningen, A. Deusinglaan 1, 9713 AV Groningen, The Netherlands; 3 DWI Leibniz-Institute for Interactive Materials, RWTH Aachen University, Forckenbeckstr. 50, 52056 Aachen, Germany; moeller@dwi.rwth-aachen.de

**Keywords:** polyelectrolyte, gel, electrodeposition, interface, electrostatic interactions, thin films, hydrodynamic voltammetry

## Abstract

The effect of polyelectrolyte composition on the electrodeposition onto platinum is investigated using a counterion switching approach. Film formation of preformed polyelectrolytes is triggered by oxidation of hexacyanoferrates(II) (ferrocyanide), leading to polyelectrolyte complexes, which are physically crosslinked by hexacyanoferrate(III) (ferricyanide) ions due to preferential ferricyanide/polycation interactions. In this study, the electrodeposition of three different linear polyelectrolytes, namely quaternized poly[2-(dimethylamino)ethyl methacrylate] (i.e., poly{[2-(methacryloyloxy)ethyl]trimethylammonium chloride}; PMOTAC), quaternized poly[2-(dimethylamino)ethyl acrylate] (i.e., poly{[2-(acryloyloxy)ethyl]trimethylammonium chloride}; POTAC), quaternized poly[*N*-(3-dimethylaminopropyl)methacrylamide] (i.e., poly{[3-(methacrylamido)propyl]trimethylammonium chloride}; PMAPTAC) and different statistical copolymers of these polyelectrolytes with *N*-(3-aminopropyl)methacrylamide (APMA), are studied. Hydrodynamic voltammetry utilizing a rotating ring disk electrode (RRDE) shows the highest deposition efficiency *DE* for PMOTAC over PMAPTAC and over POTAC. Increasing incorporation of APMA weakens the preferred interaction of the quaternized units with the hexacyanoferrate(III) ions. At a sufficient APMA content, electrodeposition can thus be prevented. Additional electrochemical quartz crystal microbalance measurements reveal the formation of rigid polyelectrolyte films being highly crosslinked by the hexacyanoferrate(III) ions. Results indicate a different degree of water incorporation into these polyelectrolyte films. Hence, by adjusting the polycation composition, film properties can be tuned, while different chemistries can be incorporated into these electrodeposited thin hydrogel films.

## 1. Introduction

Electrodeposition is a powerful and easily controllable tool for triggering film formation by imposing an electrical signal. The possibility of electrodepositing inter alia polymers [1,2], metals [3] or polymer-metal compound composites [4,5,6] onto different substrates [7,8,9,10,11,12] provides access to a wide variety of functional films. These films offer exciting applications in material science [13], the biomedical field [8,14,15,16], sensing [16,17], and electronics [18]. The incorporation of stimulus sensitivity, e.g., the change of properties upon application of an external trigger (voltage, pH, temperature, light, etc.) is an appealing way to generate smart polymer systems [19,20,21]. Voltage is used as an external trigger in electrochemical film formation, provoking deposition of the investigated system onto the electrode. Although electroactive monomers/polymers are often used for electrodeposition [22,23], this process is not limited to electroactive polymeric compounds. Polyelectrolytes, for instance, are often deposited in an electrochemical manner utilizing the possible complex formation of these polymers with oppositely charged compounds. In this context, a common way of depositing weak, non-electroactive, polyelectrolytes exploits an electrochemically provoked pH change close to the electrode. This pH change can induce either precipitation by discharging the polyelectrolytes [24,25] or enabling complexation and subsequent precipitation with other polyelectrolytes or multivalent ions upon charging [1,2]. Another way of introducing electrochemical stimulation in those systems is the complex formation of polyelectrolytes with electroactive counterions [26,27]. As in previous works, we use this modular approach to perform a counterion valency-induced deposition (CVID) of cationic polyelectrolytes interacting with hexacyanoferrate ions [28]. Film formation and film dissolution triggered by a valency change of the hexacyanoferrate redox couple has already been known since the 1980s. Anson et al. were the first to report on the electrodeposition of insoluble ferricyanide (hexacyanoferrate(III), [Fe(CN)_6_]^3−^, with HCF(III) as abbreviation)–polycationic siloxane complexes onto electrodes from solutions of soluble ferrocyanide (hexacyanoferrate(II), [Fe(CN)_6_]^4−^, with HCF(II) as abbreviation)–polycationic siloxane complexes. Anodic oxidation allows for film formation by producing ferricyanide ions that physically crosslink the polycationic species towards a film, which is highly transparent to electrons (electron transfer kinetics is not governed by charge transport within the film) [27,29]. The pronounced interaction with the less charged ferricyanide ions was found for many polycationic polymers, indicating enthalpic contributions to be dominating. Especially polyelectrolytes containing quaternary amine functions were found to show the described electrochemically modifiable interaction pattern with the hexacyanoferrate redox couple [27,29,30,31,32].

In a previous study, we investigated different architectures of the cationic poly{[2-(methacryloyloxy)ethyl]trimethylammonium chloride} (PMOTAC) and their influence on electrodeposition using the CVID approach [28]. The architecture was varied between monomer units, linear and star-shaped polymers, and crosslinked polymer particles (microgels). Film deposition was only observed for linear and star-shaped polymers. Further quantification of the deposition was achieved for the hydrodynamic voltammetry measurements with a rotating ring disk electrode (RRDE) by implementing the deposition efficiency *DE*. The *DE* assigns the ratio of charge used to produce the deposited electroactive species to the total charge. Thus, we could derive that linear polymers of moderate mass are most efficient with respect to film formation enabled by the intermolecular physical crosslinks of the ferricyanide ions. Intramolecular crosslinking becomes more favourable upon branching and increasing the molecular mass. For microgel particles, intramolecular crosslinking is predominant, and deposition of this highly crosslinked particles is thus prevented.

**Scheme 1:**
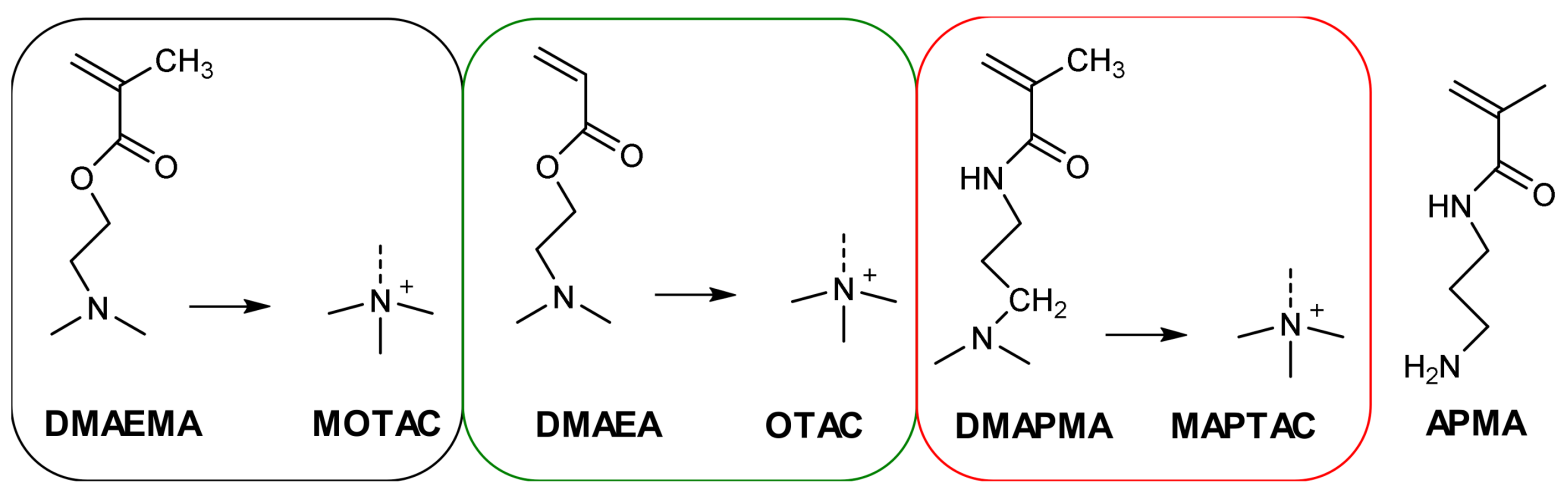
Chemical structures of the used monomers including the abbreviations (DMAEMA: 2-(dimethylamino)ethyl methacrylate; MOTAC: [2-(methacryloyloxy)ethyl] trimethylammonium chloride; DMAEA: 2-(dimethylamino)ethyl acrylate; OTAC: [2-(acryloyloxy)ethyl]trimethyllammonium chloride; DMAPMA: 3-(dimethylamino)propyl methacrylamide; MAPTAC: [3-(methacrylamido)propyl]trimethylammonium chloride; APMA: *N*-(3-aminopropyl) methacrylamide) of the respective quaternized compounds.

In this manuscript, we further investigate the tunability of the polyelectrolyte electrodeposition by studying three linear cationic polymers of different hydrophilicity: PMOTAC, poly{[2-(acryloyloxy)ethyl]trimethylammonium chloride} POTAC (quaternized poly[2-(dimethylamino)ethyl acrylate] *q*PDMAEA) and poly{[3-(methacrylamido)propyl]trimethylammonium chloride} PMAPTAC (Scheme 1). Additionally, these systems are copolymerized with the monomer *N*-(3-aminopropyl)methacrylamide hydrochloride (APMA) up to 30% content of APMA. Incorporation of this comonomer enhances the electrodeposition tunability. Further, the reactive amine function of APMA provides the opportunity for post-modification.

## 2. Materials and Methods

The monomers 2-(dimethylamino)ethyl methacrylate (DMAEMA) and *N*-[3-(dimethylamino)propyl] methacrylamide, *N*-(3-aminopropyl)methacrylamide hydrochloride (APMA), potassium hexacyanoferrate(II) trihydrate (K_4_[Fe(CN)_6_]·3H_2_O and methyl iodide were purchased from Sigma-Aldrich (Taufkirchen, Germany), 2-(Dimethylamino)ethyl acrylate (DMAEA) and dimethylformamide (DMF, low in water (<150 ppm) from VWR (Langenfeld, Germany), 2,2′-azobis(2-methylpropionitril) (AIBN) from Fluka, potassium chloride and potassium hexacyanoferrate(III) (K_3_[Fe(CN)_6_]·3H_2_O from Merck (Darmstadt, Germany). D_2_O was purchased from Deutero (Kastellaun, Germany). All chemicals were used as received without further purification. Water was deionized by an Astacus² apparatus (0.056 µS).

The polymers were synthesized in a free radical polymerization in DMF. A typical example is given for P(MOTAC-*co*-APMA-15%); the other polymers were synthesized accordingly. The initiator AIBN (0.054 g, 0.3 mmol) was dissolved in 3 mL DMF and degassed with nitrogen for 30 min. The monomers 2-(dimethylamino)ethyl methacrylate (DMAEMA) (2.93 g, 18.7 mmol) and *N*-(3-aminopropyl)methacrylamide hydrochloride (APMA) (0.58 g, 3.3 mmol, 15 mol % of total monomer amount) were dissolved in 22 mL DMF and degassed with nitrogen for 30 min. The monomer solution was tempered in an oil bath at 78 °C for 20 min before the initiator solution was added using a syringe. The reaction was terminated after 24 h in an ice bath. Methyl iodide (5.30 g, 37.3 mmol) was added in excess to the reaction mixture. The solution was allowed to warm up to room temperature and stirred overnight. For purification and ion exchange, the solution was dialyzed (MWCO 1000 Da; regenerated cellulose) against a 2 M sodium chloride solution, a 0.1 M sodium chloride solution and against purified water in each case for 24 h stirred at room temperature. In some cases, where iodide removal was only partial (as seen in another plateau in the hydrodynamic voltammograms), the polymers were again dissolved in approximately 30 mL water. Then, a spoon full of sodium chloride was added and the solutions were dialyzed against water over several days before lyophilization.

^1^H-NMR spectra were recorded from 10–20 mg/mL polymer solution in D_2_O with a 400-MHz Bruker DRX 400 NMR spectrometer (Bruker, Rheinstetten, Germany) at room temperature. The chemical shifts are indicated in parts per million downfield from the TMS standard using the D_2_O signal as reference.

Size exclusion chromatography measurements were performed with 3–4 g/L polymer solutions in 0.1 M NaCl, 0.3 vol % trifluoroacetic acid (TFA), 0.01% NaN_3_ using a refractive index detector (Jasco RI-2031, Jasco, Pfungstadt, Germany). Molecular weights (*M_n_*, *M*_w_) were determined using cationic poly(2-vinylpyridine) P2VP (in 0.1 M NaCl, 3% TFA) as calibration.

Electrochemical measurements were performed on the CH Instruments Electrochemical Workstation Potentiostat CHI760D (Austin, TX, USA). A conventional three-electrode setup at room temperature was used if not stated otherwise. In all cases, a spirally platinum electrode (23 cm length) and an Ag/AgCl electrode stored in 1 M KCl served as aqueous counter and reference electrode respectively. All solutions were degassed with argon prior to measurements.

For hydrodynamic voltammetry, a rotating ring disk electrode was used as working electrode exhibiting a platinum disk and ring of 4 mm disk diameter and 7 mm/5 mm outer/inner ring diameter. Hydrodynamic voltammograms were measured of 0.13 mM [Fe(CN)_6_]^4−^ in 0.1 M KCl in absence and presence of different polyelectrolytes at room temperature (forward and backward scanning of the potential between 0 and 0.5 V vs. Ag/AgCl). For an initial charge ratio (*icr*) between counterion to polyelectrolyte of 1, 0.52 mM polyelectrolyte (with respect to quaternized units) was used. The *icr* value was varied by keeping the [Fe(CN)_6_]^4−^ concentration constant while changing the polyelectrolyte concentration to adjust *icr* values of 0.5, 0.9, 1, 1.1, 1.5 and 2.0. The rotation rate was varied between 200–5000 rpm.

Electrochemical quartz crystal microbalance measurements were conducted with a QCM200 Quartz Crystal Microbalance from Stanford Research Systems SRS. An AT-cut quartz crystal (5 MHz) from SRS (distributor: BELLTEC, Wesel, Germany) coated with a Ti/Pt electrodes (exposed area of the front electrode in contact with the liquid ~1.37 cm^2^) was used as working electrode. Measurements of 0.52 mM polymer, 0.13 mM [Fe(CN)_6_]^4−^ in 0.1 M KCl were performed in a water-jacket cell at 20 °C. Changes in frequency and resistance when applying a voltage (sweep) were measured.

Potentiometric Titration measurements were performed on a Methrohm 665 autotitrator (Metrohm, Filderstadt, Germany). Titration was performed for pure 0.1 M KCl solution (78.9 mL) and 10.1 mg PAPMA in 0.1 M KCl solution (83.8 mL). Both solutions were acidified with 0.1 M HCl to pH 3 before 0.1 M NaOH was added in 2 µL steps. The resulting pH values were compared with respect to the relative volume of NaOH added.

## 3. Results

The polymers are synthesized in a free radical copolymerization of the corresponding uncharged monomers 2-(dimethylamino)ethyl methacrylate (DMAEMA), 2-(dimethylamino)ethyl acrylate (DMAEA) and 3-(dimethylamino)propyl methacrylamide (DMAPMA) to prevent any charge-induced complications during polymerization of the corresponding quaternized monomers (Scheme 1). For copolymerization, the molar amount of APMA in the synthesis is varied from 0% to 30%. Full quaternization of the DMAEMA, DMAEA und DMAPMA units is performed by addition of methyliodide without an additional base (Supporting Information Figures S1–S3; Scheme S1 and S2). Under these conditions, the primary amine function of the APMA moiety is rather unreactive and can be methylated only once towards a still-reactive secondary amine function (Supporting Information Figure S7 [33]. Incorporation of APMA can be proven by ^1^H-NMR (Supporting Information Figures S4–S6, Table S1). In addition, pure poly[*N*-(3-aminopropyl) methacrylamide] PAPMA, which has been synthesized by free radical polymerization, is also addressed in this work (Supporting Information). Earlier works have shown that the molecular weight influences the deposition behavior. Nevertheless, increasing the molecular weight by more than one order of magnitude reduces the *DE* only by 25% [28]. As the polymers discussed in this paper show only a rather small variation in the degree of polymerization *P*_n_ (typical *P*_n_ variation not more than a factor of 2; see Supporting Information for details), there is no need for a better control in polymerization, which would have been available for, e.g., controlled radical polymerization (Supporting Information Tables S1 and S2, which show a relatively narrow molar mass distribution, based on size exclusion chromatography).

**Figure 1:**
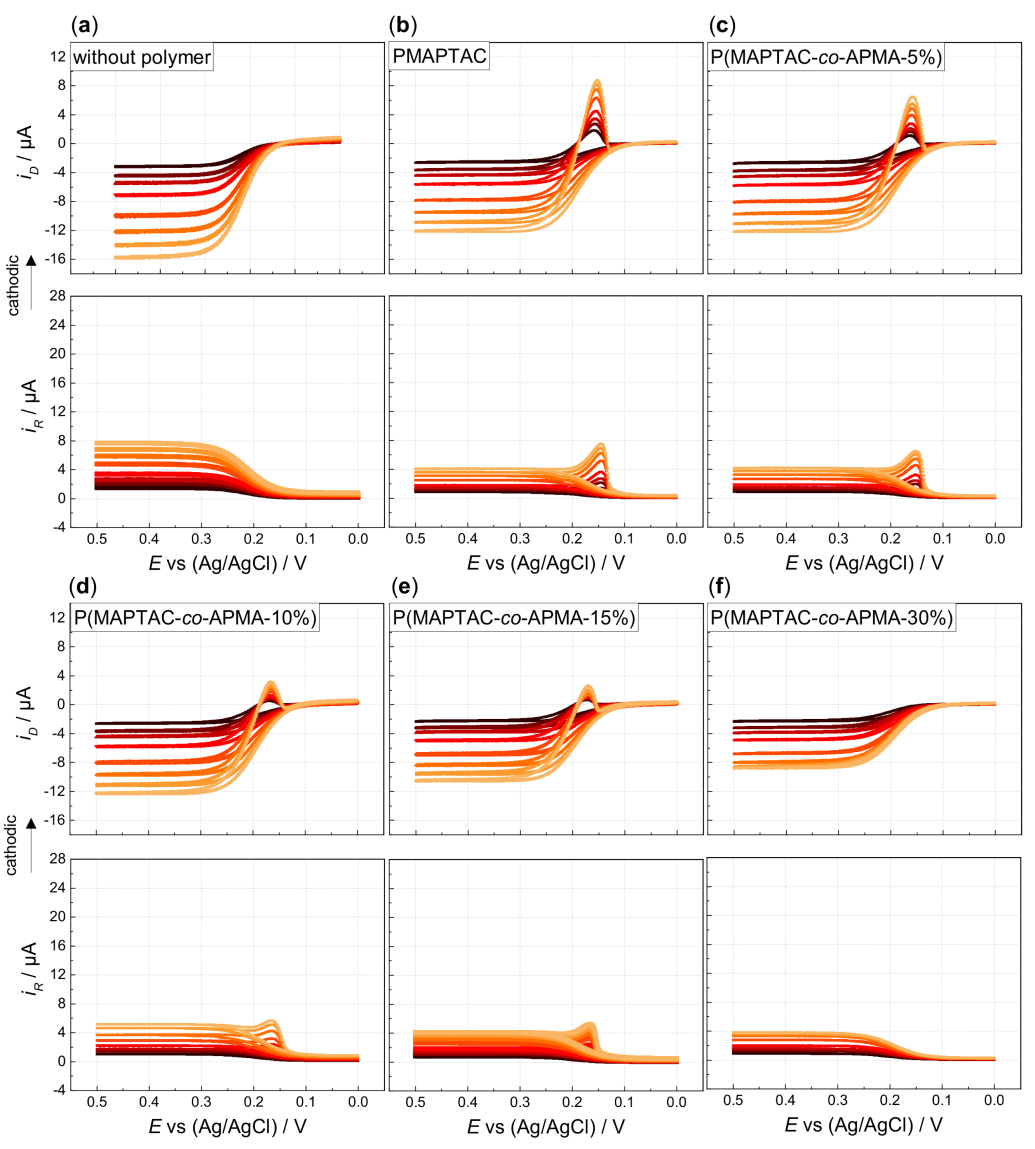
Hydrodynamic voltammograms of 0.13 mM HCF(II) in 0.1 M KCl in (**a**) absence and presence of 0.52 mM (with respect to quaternized polymer units, *icr* = 1); (**b**) PMAPTAC; (**c**) P(MAPTAC-*co*-APMA-5%); (**d**) P(MAPTAC-*co*-APMA-10%); (**e**) P(MAPTAC-*co*-APMA-15%) and (**f**) P(MAPTAC-*co*-APMA-30%) (ring potential at 0 V vs. Ag/AgCl, disk potential scanned from 0 to 0.5 V (forward scan) and back to 0 V (backward scan) vs. Ag/AgCl with *ν* = 5 mV/s, rotation rates increase as indicated from black to orange: 200, 400, 600, 1000, 2000, 4000, 5000 rpm).

**Figure 2:**
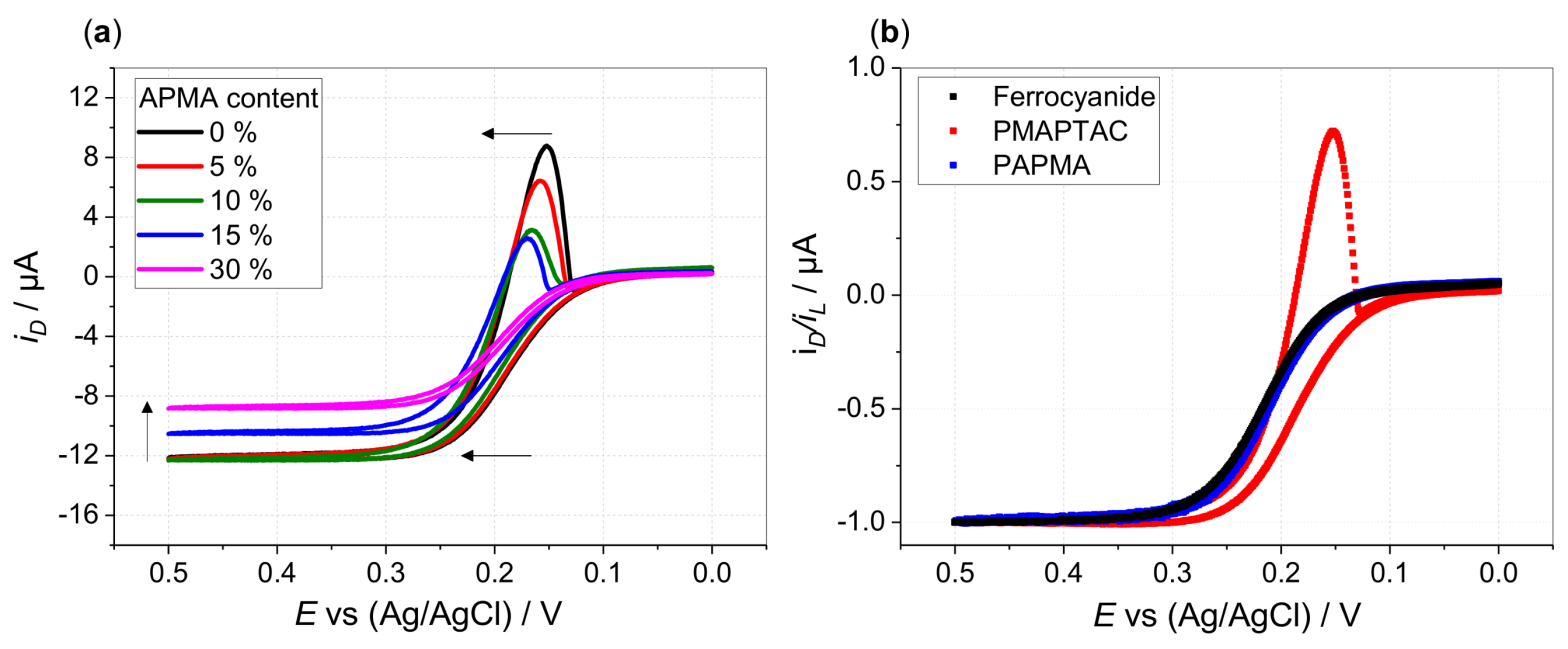
(**a**) Disk currents at 5000 rpm in hydrodynamic voltammograms of 0.13 mM HCF(II) in 0.1 M KCl (black line) in presence of 0.52 mM P(MAPTAC-*co*-APMA-x%) (with respect to quaternized units, *icr* = 1) with x% APMA content as indicated: 0% (black), 5% (red), 10% (green), 15% blue and 30% magenta (arrow indicates shift of voltammograms with increasing APMA content) and (**b**) disk currents normalized to limiting disk currents for hydrodynamic voltammograms of 0.13 mM HCF(II) in 0.1 M KCl in absence (black) and presence of 0.52 mM PMAPTAC (red) and PAPMA (blue) (with respect to quaternized units, *icr* = 1) (disk potential scanned from 0 to 0.5 V (forward scan) and back to 0 V (backward scan) vs. Ag/AgCl with *ν* = 5 mV/s).

Hydrodynamic voltammetry is the major method used in this work to evaluate the deposition performance of the polymer systems. A rotating ring disk electrode (RRDE) is used to measure the electrochemical behavior of the different hexacyanoferrate/polymer solutions. The disk potential is swept from 0 to 0.5 V (forward scan) and back to 0 V (backward scan) vs. Ag/AgCl while the ring potential remains constant at 0 V vs. Ag/AgCl. Starting from a solution containing solely ferrocyanide, these ions are oxidized to ferricyanide at sufficiently high potential within the forward scan (Figure 1a). Increasing the rotation rate results in an enhanced mass transport to the electrode and thus in enhanced currents because of a thinner diffusion layer at the electrode surface [34]. Currents are reduced upon addition of polyelectrolytes, proving some attraction between the quaternized units and the oppositely charged hexacyanoferrates (here ferrocyanide). Due to this attraction, the multivalent ferrocyanide is (partly) coupled to the polyelectrolyte scaffold. The smaller (apparent) diffusion coefficients derived from Levich plots confirm slower diffusion/reduced availability of the ferrocyanide upon polymer addition (Supporting Information Figure S11). More importantly, the voltammograms shift towards more negative potentials upon addition of the polyelectrolytes, due to the preferred interaction of the quaternized units with the ferri- and not the ferrocyanide (Figure 2b) [35]. Hence, the oxidation of ferro- to ferricyanide strengthens the interaction pattern of these two oppositely charged components in solutions of hexacyanoferrates and the corresponding quaternized polymeric sites. As discussed, the less-charged ferricyanides form an insoluble complex with the quaternized units of the polyelectrolyte (facilitated by ion-specific effects), resulting in film formation onto the electrode. Here, the change in counterion charge does not alone explain the decreased solubility in presence of ferricyanide, though CVID still holds true. Then, the film is held together by bridging ferricyanides, physically crosslinking the monomer moieties via the quaternized ammonium groups. Due to the chemical reversibility of the system, any ferricyanide, which was trapped and stored at the electrode, can be reduced back to ferrocyanide at a sufficiently low potential within the backward scan. These ferricyanides, being reduced rather instantaneously, give rise to a cathodic peak in disk current during this reverse scan (Figure 1b for exemplary PMAPTAC, Supporting Information Figures S9 and S10). However during film dissolution, some parts of the film are swept away prematurely to full reduction of the incorporated ferricyanides that are then partially reduced at the ring electrode. This results in a cathodic peak also being visible in the detected ring currents of the RRDE.

Comparing copolymers with increasing APMA content, the cathodic peaks diminish till the absence of any peak indicates prevention of deposition (30% APMA content in case of P(MAPTAC-*co*-APMA); see Figure 1c–f). The cathodic peaks not only decrease, they also show less extension towards negative potentials. Film dissolution is completed at higher positive potentials. Concomitantly, the increasing APMA content causes a shift of the voltammograms towards more positive potentials and a decrease of the limiting currents (Figure 2a). The copolymer system “prefers” less and less the interaction with the ferri-compared to the ferrocyanide. Indeed, the voltammogram of HCF(II) in the presence of pure PAPMA homopolymer does not exhibit any shift in potentials compared to a pure ferrocyanide solution (Figure 2b, Supporting Information Figures S12 and S13). Unlike the quaternized amine groups, the primary/secondary amine groups of APMA do not show the ion-specific interaction that causes the quaternized units to interact preferentially with the less charged ferricyanide ions. When starting from a solution of ferricyanide and PAPMA, deposition occurs by scanning the potential from 0.5 to 0 V and back to 0.5 V vs. Ag/AgCl (Supporting Information Figure S14). Thereby, interaction of the primary/secondary amine functions of the APMA units is enabled by pH-dependent charging as shown by potentiometric titration (Supporting Information Figure S8). Under typical conditions for electrodeposition (pH ranges from pH 6 for 0.1 M KCl + added strong polyelectrolytes PMOTAC, POTAC and PMAPTAC to pH 8 for 0.1 M KCl + 0.52 mM of pure PAPMA as a weak polyelectrolyte), basically all monomeric units are charged. This allows the preferential electrostatic interaction of PAPMA with ferrocyanides, as the APMA units are mainly only deprotonated above pH 8. Otherwise, the quaternized ammonium groups of the strong polyelectrolytes do not change their charge with pH anyway. This specific interaction pattern with primary amine groups was also found in literature, where polyelectrolyte multilayers comprising poly(allylamine hydrochloride) (PAH) were found to be stronger crosslinked by ferro- than by ferricyanide [36]. Moreover, deposition of PAH–ferrocyanide films onto gold electrodes was shown to be possible by reducing ferri- to ferrocyanide [37]. This indicates the interaction of the hexacyanoferrate redox couple differs for quaternary and primary amine functions. This is supported by the larger apparent diffusion coefficient of ferricyanide in the presence of PAPMA (3.74 ± 0.05 × 10^−6^ cm^2^/s as derived via the Levich equation) compared to the diffusion coefficient of ferrocyanide in the presence of PAPMA (1.85 ± 0.02 × 10^−6^ cm^2^/s). However, within this context, it is conceivable that the former clear solutions of PAPMA (or copolymers with high APMA content) turn turbid upon ferrocyanide addition. An entangled/aggregated network is formed by the ferrocyanide ions due to physical crosslinking of the primary/secondary amine functions of the APMA units. The competition of the quaternized units and the APMA units for the stronger crosslinking hexacyanoferrate ion prevents deposition above a certain APMA content. This lack of deposition could be even facilitated by the above-mentioned preaggregation (higher molar mass).

**Figure 3:**
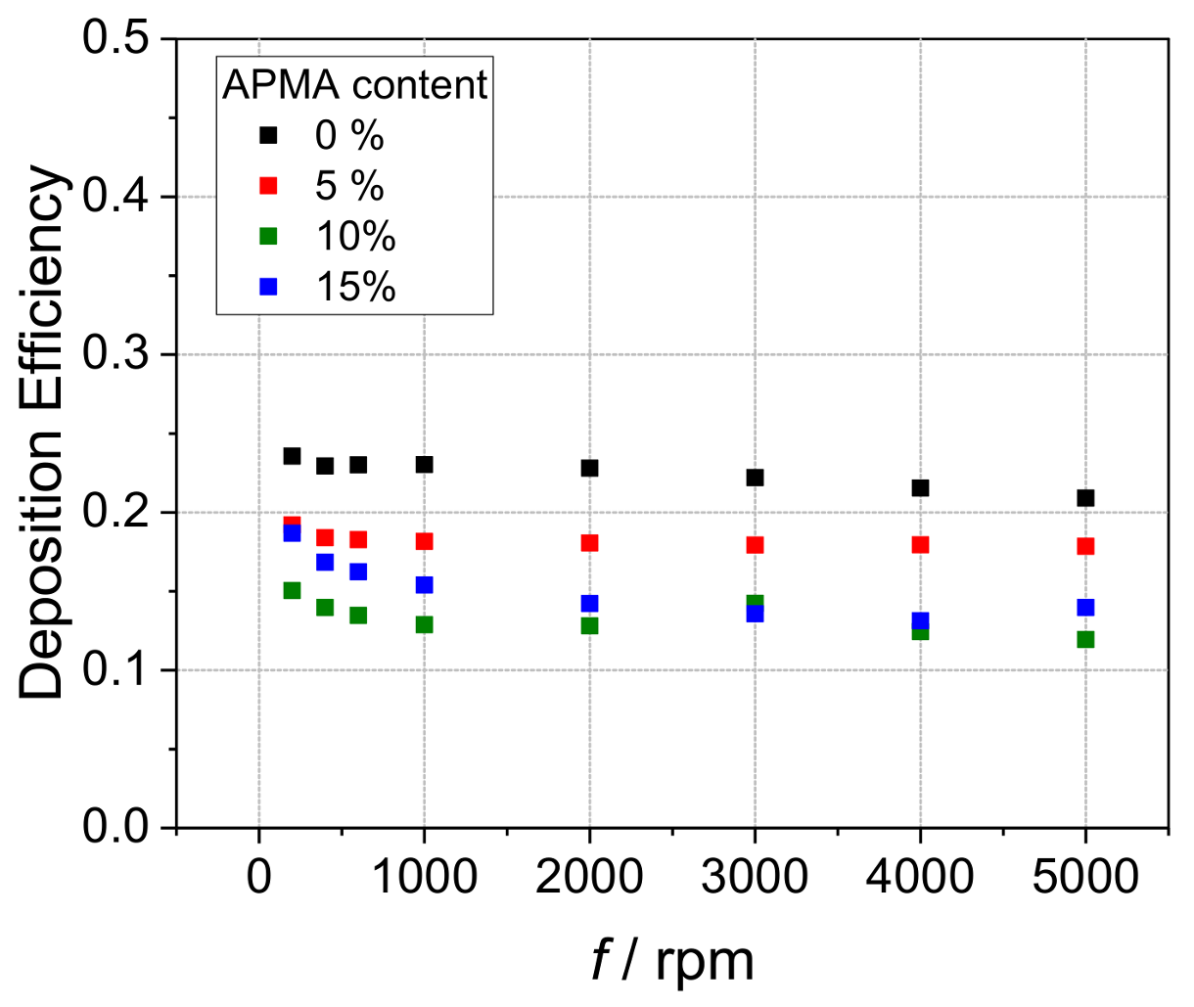
Deposition efficiencies vs. rotation rate *f* in hydrodynamic voltammograms of 0.13 mM HCF(II) in 0.1 M KCl in presence of 0.52 mM (with respect to quaternized monomer units, *icr* = 1) for P(MAPTAC-*co*-APMA-x%) with 0% (black square), 5% (red square), 10% (green square) and 15% (blue square) APMA content.

Performing hydrodynamic voltammetry with a RRDE allows for quantification by different parameters. The so-called collection efficiency *N*, the ratio of ring to disk limiting current, is one of such deducible values. This parameter, which defines how much of the disk-generated ferricyanide is detected at the ring electrode, is clearly reduced in the presence of the investigated polycationic species as a result of complexation and deposition (Supporting Information Figure S15) [28,34]. Another value is the deposition efficiency *DE* that assigns the ratio of charge used to produce the deposited electroactive species to the total charge transferred (for further information view Supporting Information). In accordance with the previous discussion, the *DE* also reflects diminishing deposition with increasing APMA content (Figure 3, Supporting Information Figure S16).

**Figure 4:**
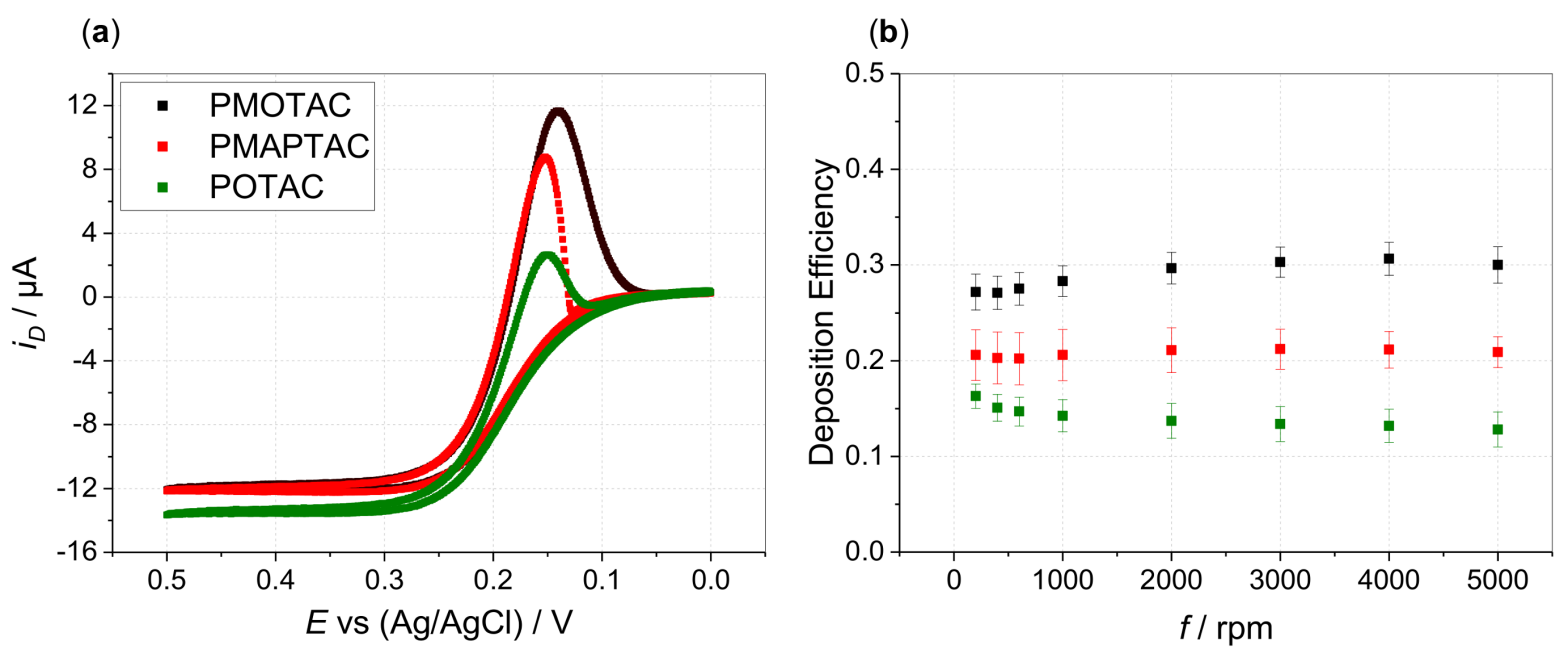
(**a**) Disk currents in hydrodynamic voltammograms at 5000 rpm (disk potential scanned from 0 to 0.5 V (forward scan) and back to 0 V (backward scan) vs. Ag/AgCl with *ν* = 5 mV/s) and (**b**) Deposition efficiency vs. rotation rate *f* of 0.13 mM HCF(II) in 0.1 M KCl in presence of 0.52 mM PMOTAC (black), PMAPTAC (red), POTAC (green) (with respect to quaternized units, *icr* = 1). *DE* values are averaged over six measurements of freshly prepared solutions (error bars show the standard deviation of the shown mean values).

Apart from the influence of increasing APMA content, comparison of the homopolymers reveals that film formation is most pronounced for PMOTAC (Figure 4a). Films formed by anodic electrodeposition from ferrocyanide—PMOTAC solutions show highest stability compared to PMAPTAC and POTAC. The cathodic peak is extended towards more negative potentials for disk and ring currents (Figure 4b, Supporting Information Figures S17 and S18). However, no remarkable difference can be seen between the different homopolymers when studying the influence of the initial charge rati*o* (*icr*) between counterion to polyelectrolyte charges (Supporting Information Figure S19). As in our previous work, stoichiometric *icr* values close to 1 result in the largest deposition efficiencies for all three polymer types. This implies also rather similar nominal charge ratios (*ncr*)/stoichiometry within the electrodeposited films. Decreasing and increasing the *icr* values suppresses electrodeposition. Hence, these results give no indication of different *ncr* in the different ferricyanide–polymer complexes. Therefore, the apparent differences in the deposition performance are more likely linked to the different hydrophilicities of the polymers. Drawing from the chemical structure, the OTAC unit shows highest hydrophilic character due to the least methyl/en groups being present. The higher hydrophilic character of the amide function [38] might not be compensated for by the additional methylene group of the MAPTAC unit compared to the MOTAC unit. This would cause the MOTAC unit to show the highest hydrophobic character, promoting both the insolubility of the ferricyanide–PMOTAC complexes for enhanced film formation and film stability due to enhanced hydrophobic interactions.

**Figure 5:**
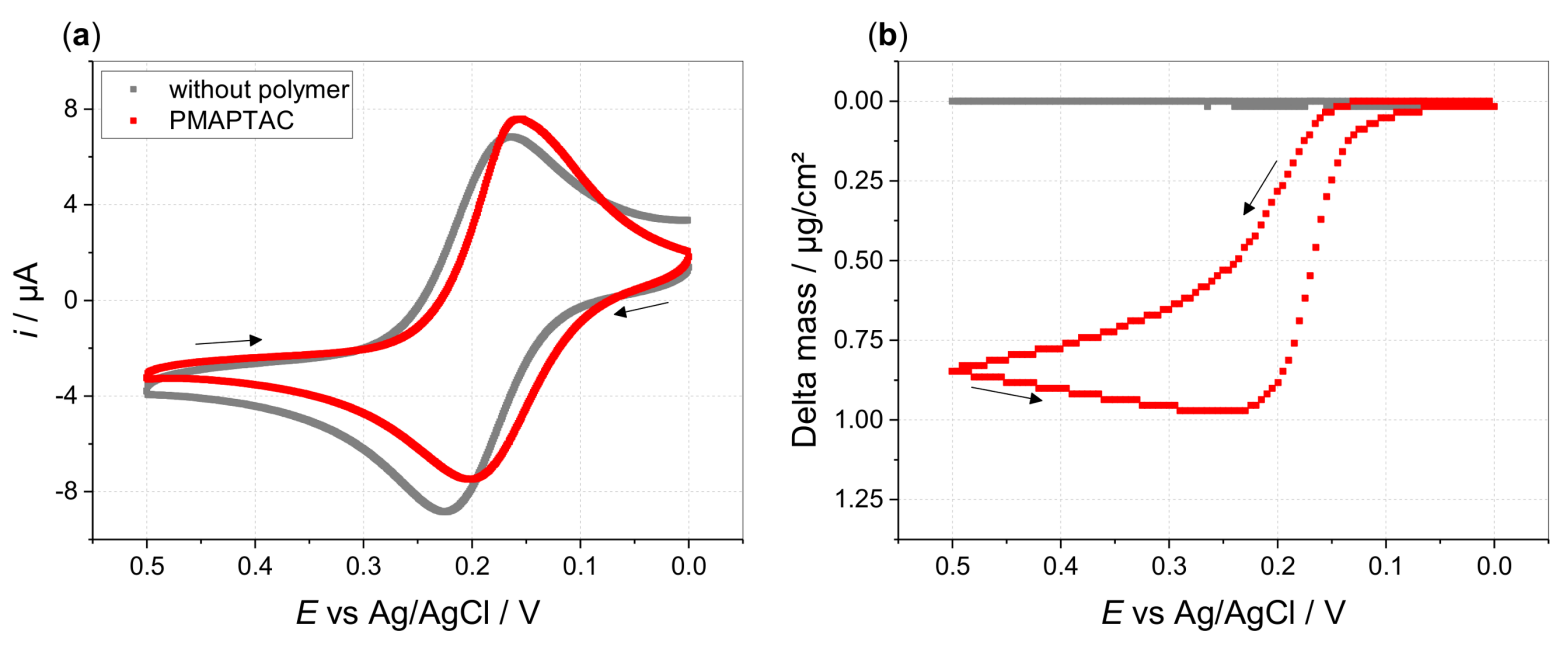
Electrochemical quartz crystal microbalance measurements during a voltage sweep (potential scanned from 0 to 0.5 to 0 V vs. Ag/AgCl with *ν* = 5 mV/s) applied to 0.1 M KCl, 0.13 mM HCF(II) in absence (grey) and presence of 0.52 mM PMAPTAC (red), (**a**) cyclic voltammograms and (**b**) delta mass vs. potential.

To further characterize the deposited films, we performed electrochemical quartz crystal microbalance (EQCM) measurements. The sensitivity of the quartz crystal resonator frequency to mass changes allows to estimate the deposited mass within the limit of the Sauerbrey equation (for further information view Supporting Information) [39]. Deviations from the Sauerbrey equation, which is strictly only valid for sufficiently rigid films, can be used to derive information about the viscoelastic properties of the deposited films [40]. Independent information about viscoelastic properties can also be derived from the motional resistance of the crystal at series resonance (given in Ω). This can be regarded as part of the Butterworth–van Dyke electrical model [40], and it relates to the dissipation of oscillation energy of the (loaded) crystal, which is directly coupled to the viscoelastic properties of deposits [41]. For the investigated polymers, no significant change in the resistance can be seen during film formation and dissolution (Supporting Information Figure S20). Hence, changes in frequency cannot be traced back to changes in viscoelasticity but to changes in mass [42]. A rigid, but still water-swollen, film seems to be formed by the different polymers being highly crosslinked by the multivalent counterions to a very dense network. Cycling the potential from 0 to 0.5 V and back to 0 V vs. Ag/AgCl provokes changes in the resonance frequency. Changes in delta mass indicate film formation during oxidation of ferro- to ferricyanide and film dissolution during the respective reduction of ferri- to ferrocyanide (Figure 5, Supporting Information Figure S21). During the backward scan, film dissolution starts at more negative potentials for the films formed by PMOTAC (Supporting Information Figure S22). This confirms the highest stability seen in hydrodynamic voltammetry for the films composed of PMOTAC compared to the ones composed of PMAPTAC or POTAC. However, the largest changes in delta mass are found for PMAPTAC solutions, despite that, according to hydrodynamic voltammetry, PMOTAC shows a higher *DE* value. As shown by variation of the *icr* value, there is no indication of a varying *ncr* charge ratio of hexacyanoferrate to polyelectrolyte for the different polymer films. Furthermore, the recorded current-voltage curves show a comparable charge flow for all polymers within these measurements. Thus, the larger changes in delta mass for PMAPTAC most probably arise from enhanced water incorporation into the formed network. This is in line with the longer side chain of PMAPTAC and the higher flexibility between the crosslinking points, allowing an increased water storage, especially close to the amide group. Therefore, the PMAPTAC films act more like thin hydrogels, being still sufficiently crosslinked to show a rather rigid behavior.

## 4. Conclusions

We investigated the electrodeposition of different (*co*)polymer systems using hydrodynamic voltammetry and the electrochemical quartz crystal microbalance. The electrochemical formation of the fully rigid films depends on the hydrophilicity of the used polymer system. We found an enhanced deposition efficiency for the polymer systems containing poly{[2-(methacryloyloxy) ethyl]trimethylammonium chloride} PMOTAC compared to the more hydrophilic poly{[2-(acryloyloxy)ethyl] trimethylammonium chloride} POTAC and poly{[3-(methacrylamido)propyl]trimethyl ammonium chloride} PMAPTAC. Incorporation of *N*-(3-aminopropyl)methacrylamide hydrochloride APMA was shown to prevent electrochemical film formation when incorporated to a critical amount and starting with ferrocyanide/polymer complexes for anodic electrodeposition. Thereby, physical crosslinking of the quaternized polymer units by the ferricyanide ions is weakened by the introduction of the primary/secondary amine groups, which are stronger crosslinked by ferrocyanide ions. Taking into account that the primary/secondary amine function allows for post-modification of the deposited films, this work contributes to the development of smart polymer coatings for future applications in the fields of e.g., materials chemistry, medicine and sensing [43,44,45,46,47,48].

## Supplementary Materials

The following are available online at http://www.mdpi.com/2073-4360/10/4/429/s1.
