# Supplementary material for: Influence of Polycation Composition on Electrochemical Film Formation
Source: Polymers (Basel). 2018 Apr 12;10(4):429. doi: 10.3390/polym10040429 (PMC6415213; doi:10.3390/polym10040429)
Supplement: Supplementary file 1 [file polymers-10-00429-s001.pdf]

polymers

MDPI

Supplementary

Sabine Schneider [1], Corinna Janssen [1], Elisabeth Klindtworth [1], Olga Mergel [1,2], Martin Möller [3] and Felix Plamper [1,*]

[1] Institute of Physical Chemistry, RWTH Aachen University, Landoltweg 2, 52056 Aachen, Germany; sabine.schneider@pc.rwth-aachen.de (S.S.); corinna.janssen@rwth-aachen.de (C.J.); elisabeth.klindtworth@rwth-aachen.de (E.K.); o.mergel@umcg.nl (O.M.)

[2] Department of Biomedical Engineering-FB40, University of Groningen, University Medical Center Groningen, A. Deusinglaan 1, 9713 AV Groningen, The Netherlands

[3] DWI Leibniz-Institute for Interactive Materials, RWTH Aachen University, Forckenbeckstr. 50, 52056 Aachen, Germany; moeller@dwi.rwth-aachen.de

* Correspondence: plamper@pc.rwth-aachen.de; Tel.: +49-241-80-94750

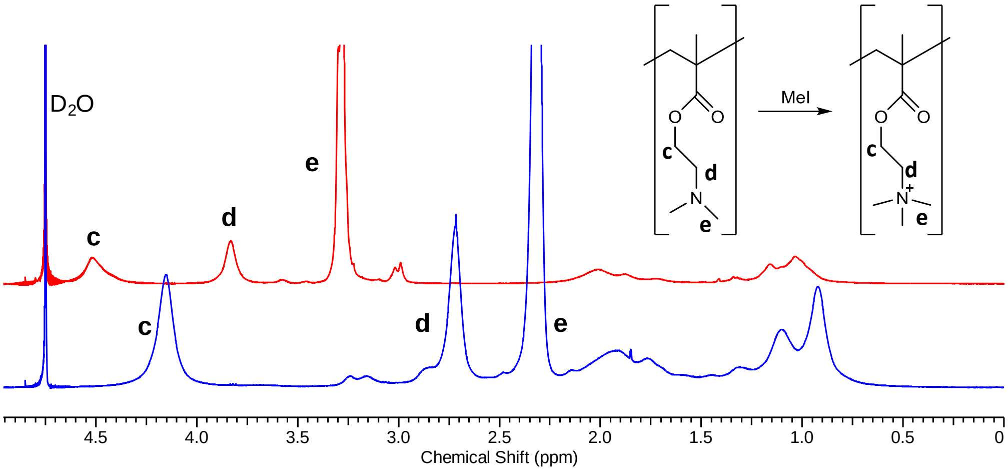

Figure S1. ^{1}H-NMR spectra in D_2O recorded for P(MOTAC-*co*-APMA-15%) before (blue) and after (red) quaternization, peaks shifting upon quaternization are assigned as indicated in the sketch at the top right.

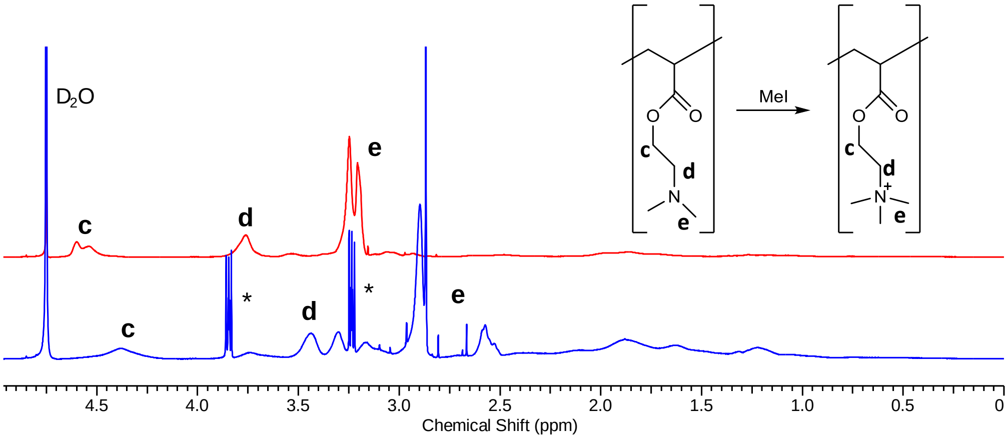

Figure S2. ^{1}H-NMR spectra in D_2O recorded for P(OTAC-*co*-APMA-15%) before (blue) and after (red) quaternization, peaks shifting upon quaternization are assigned as indicated in the sketch at the top right (* solvent impurities).

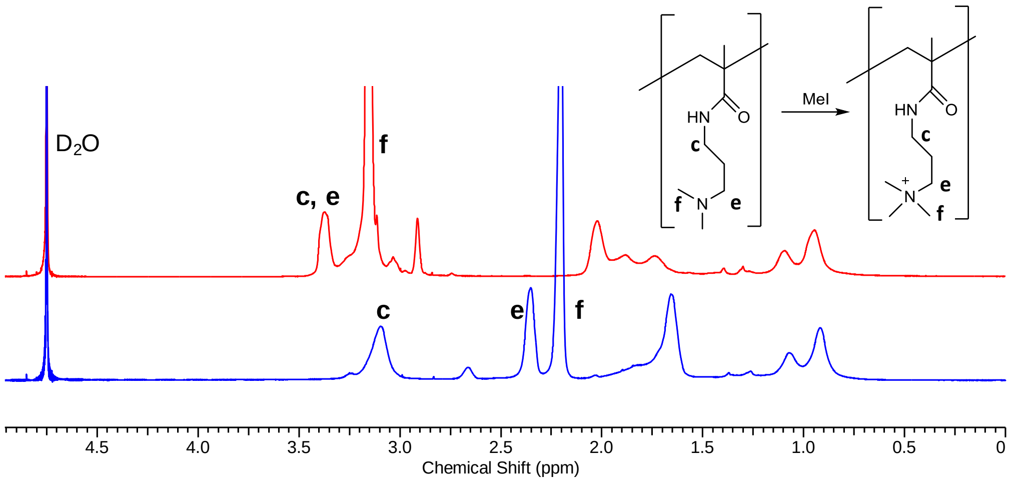

Figure S3. ^{1}H-NMR spectra in D_2O recorded for P(MAPTAC-*co*-APMA-15%) before (blue) and after (red) quaternization, peaks shifting upon quaternization are assigned as indicated in the sketch at the top right.

Characterization of Synthesized Polymers

The synthesized polymers were characterized by ^{1}H-NMR to determine the amount of APMA polymerized into the corresponding copolymer systems. Therefore, the signal of methyl groups of the quaternized units is set to 9 for the MOTAC and OTAC copolymer systems. The APMA content is determined via the integral corresponding to the methylene groups adjacent to amide and amine function of APMA (equations and schemes below, and Figures S4, S5). In the case of the MAPTAC polymer systems the signal of the methyl groups (quaternary amine function) and methylene group adjacent to the quaternary amine function overlap with the methylene group adjacent to the APMAs amine function. Thus, the signal of the methyl and methylene groups of MAPTAC and the methylene group of APMA is set to 11 + x. x corresponds to the integral value found for the APMA methylene group adjacent to the amide function (Figure S6). The APMA contents determined by this procedure are shown in Table S1. For P(MOTAC-*co*-APMA-x%), the APMA content is comparable to the initial monomer ratio used in the synthesis (5 %, 10 %, 15 % and 30 % of APMA). A larger amount is incorporated for the P(MAPTAC-*co*-APMA-x%) and P(OTAC-*co*-APMA-x%) copolymer systems.

For MOTAC and OTAC copolymer sytems:

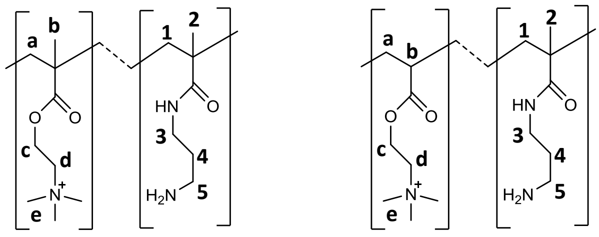

P(MOTAC-*co*-APMA-x%) P(OTAC-*co*-APMA-x%)

Scheme S1: Structure of MOTAC and OTAC copolymer systems with assignments used in ^{1}H-NMR for determination of the APMA content x_{APMA} (see equation below).

$$x_{APMA} = \frac{A_{3,5(APMA)}/H_{3,5(APMA)}}{A_{3,5(APMA)}/H_{3,5(APMA)} + A_{e(MOTAC/OTAC)}/H_{e(MOTAC/OTAC)}}$$

For MAPTAC copolymer systems:

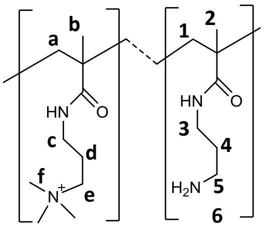

P(MAPTAC-*co*-APMA-x%)

Scheme S2. Structure of MAPTAC copolymer systems with assignments used in ^{1}H-NMR for determination of the APMA content x_{APMA} (see equation below).

$$x_{APMA} = \frac{A_{3(APMA)}/H_{3(APMA)}}{A_{3(APMA)}/H_{3(APMA)} + \left(A_{e,f,5(MAPTAC+APMA)} - A_{3(APMA)}\right)/H_{e,f(MAPTAC)}}$$

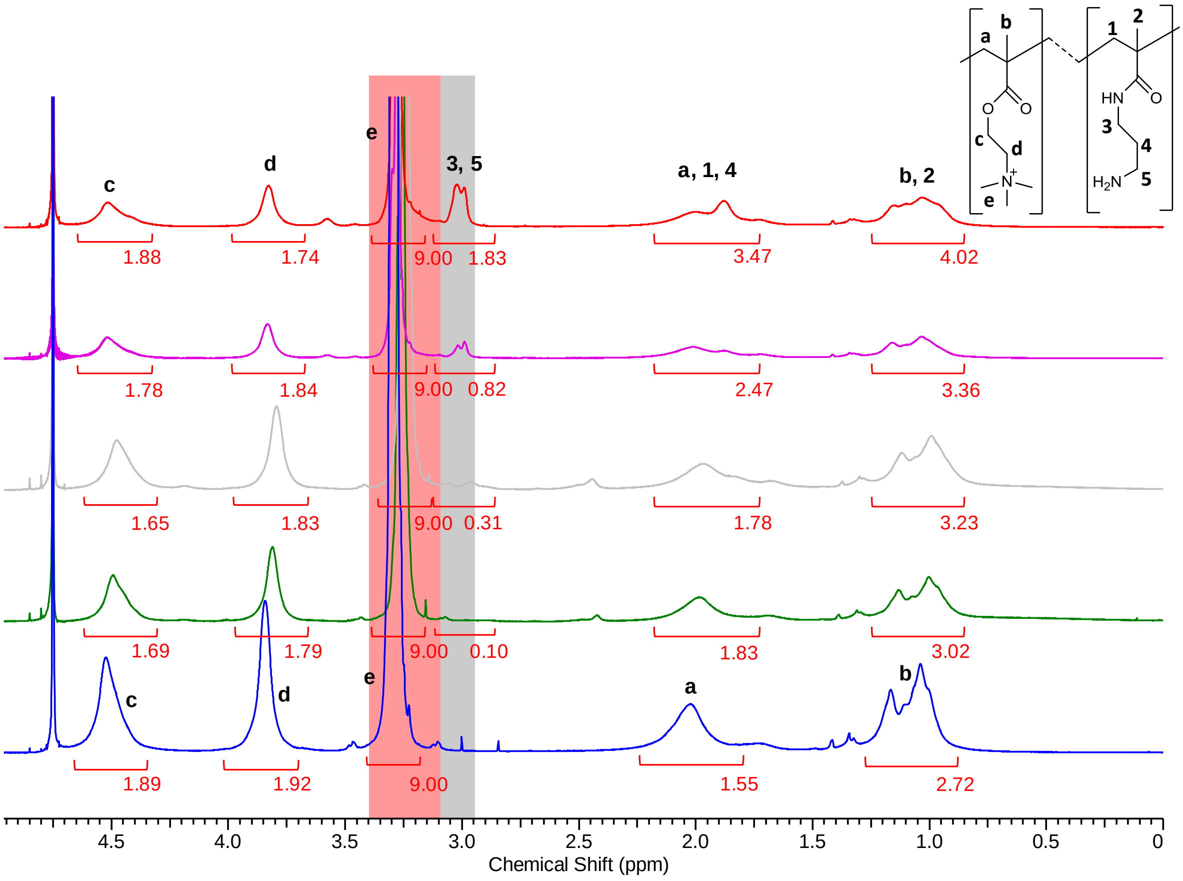

Figure S4. ^{1}H-NMR spectra in D_2O recorded for PMOTAC (blue), P(MOTAC-*co*-APMA-5%) (green), P(MOTAC-*co*-APMA-10%) (grey), P(MOTAC-*co*-APMA-15%) (magenta) and P(MOTAC-*co*-APMA-30%) (red). Red marked integral is set to 9 for spectrum 2 – 6 corresponding to 9 H-atoms (MOTAC, e) and grey marked integral is used to calculate the APMA content corresponding to 4 H-atoms from the APMA methylene groups (3, 5).

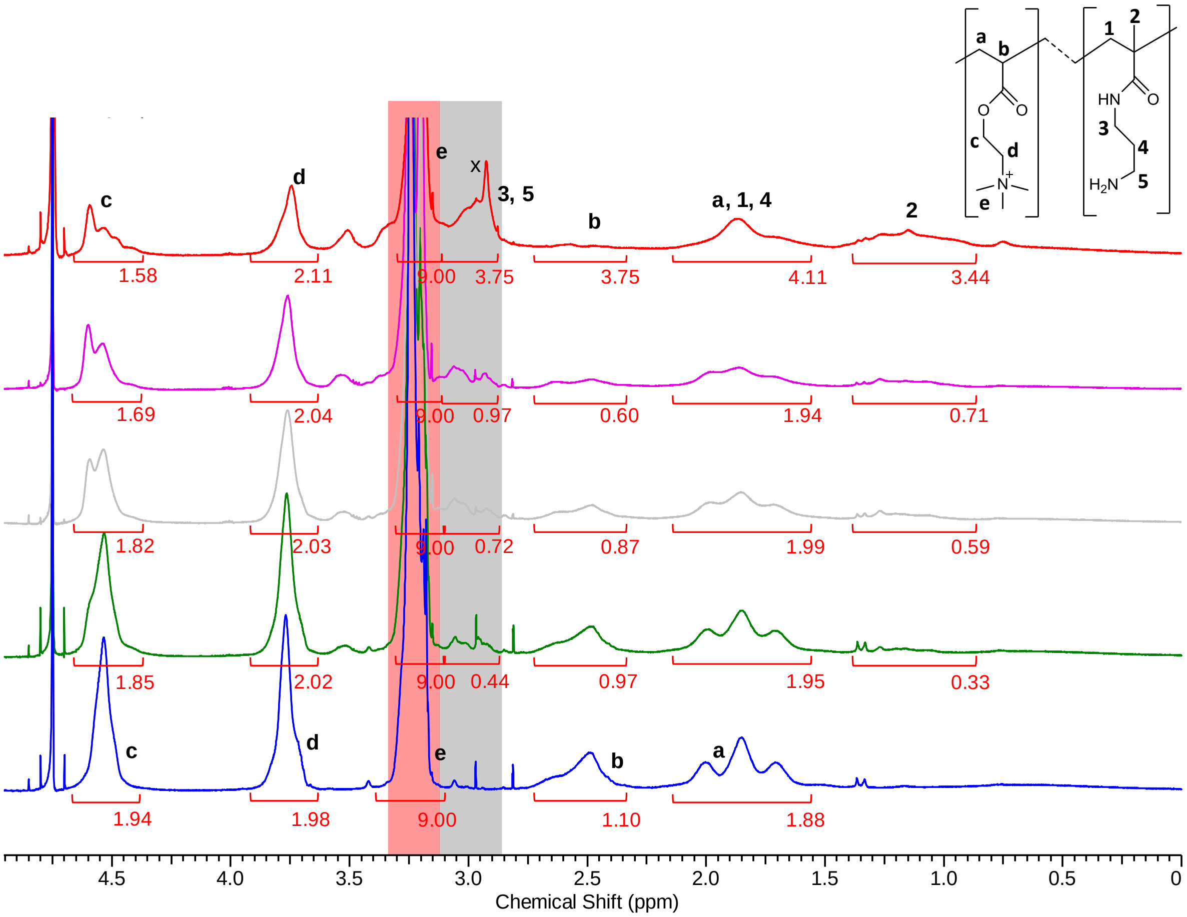

Figure S5. ^{1}H-NMR spectra in D_2O recorded for POTAC (blue), P(OTAC-*co*-APMA-5%) (green), P(OTAC-*co*-APMA-10%) (grey), P(OTAC-*co*-APMA-15%) (magenta) and P(OTAC-*co*-APMA-30%) (red). Red marked integral is set to 9 for spectrum 2 – 6 corresponding to 9 H-atoms (OTAC, e) and grey marked integral is used to calculate the APMA content corresponding to 4 H atoms from the APMA methylene groups (3, 5) (x impurities overlapping spectra and impeding determination of the APMA content).

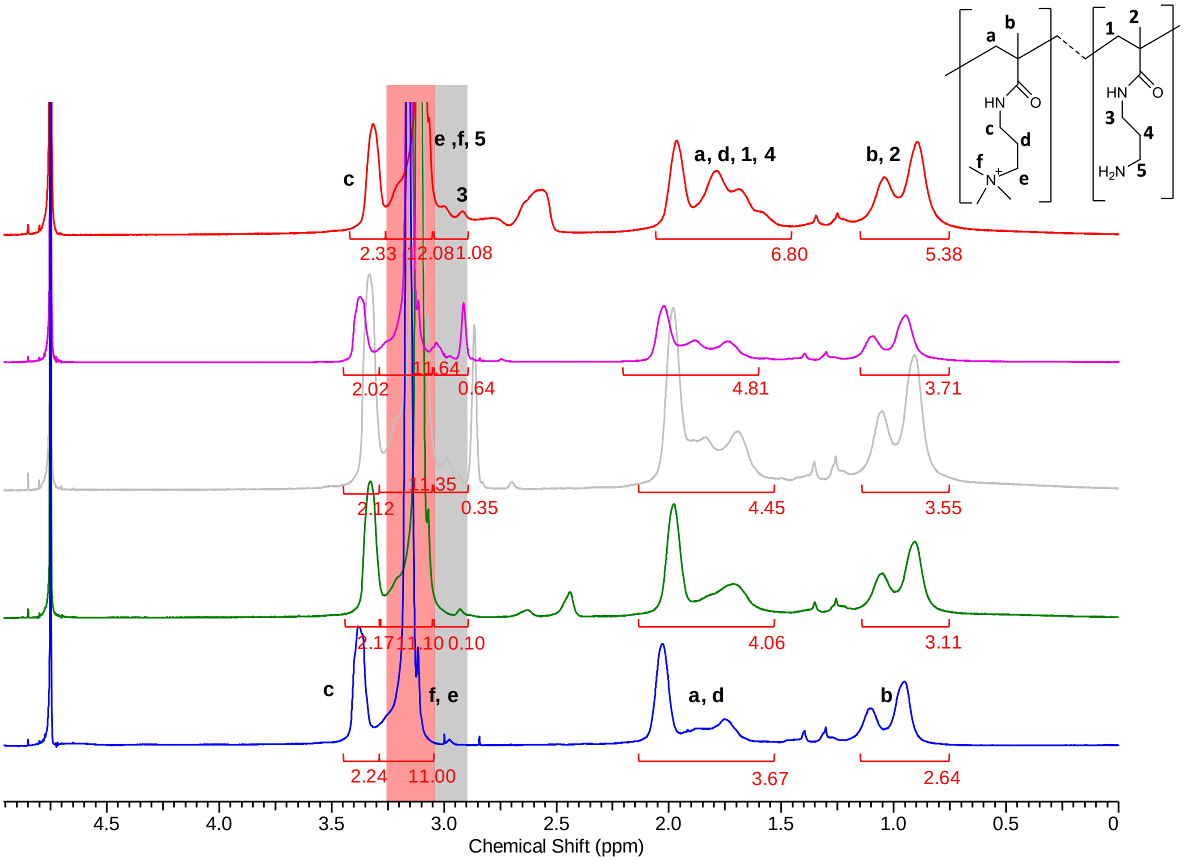

Figure S6. ^{1}H-NMR spectra in D_2O recorded for PMAPTAC (blue), P(MAPTAC-*co*-APMA-5%) (green), P(MAPTAC-*co*-APMA-10%) (grey), P(MAPTAC-*co*-APMA-15%) (magenta) and P(MAPTAC-*co*-APMA-30%) (red). Red marked integral is set to 9 + integral value of the grey marked signal for spectrum 2 – 6 corresponding to 9 (MAPTAC, e) and 2 (APMA, 5) H-atoms and grey marked integral is used to calculate the APMA content corresponding to 2 H-atoms from the APMA methylene group (3).

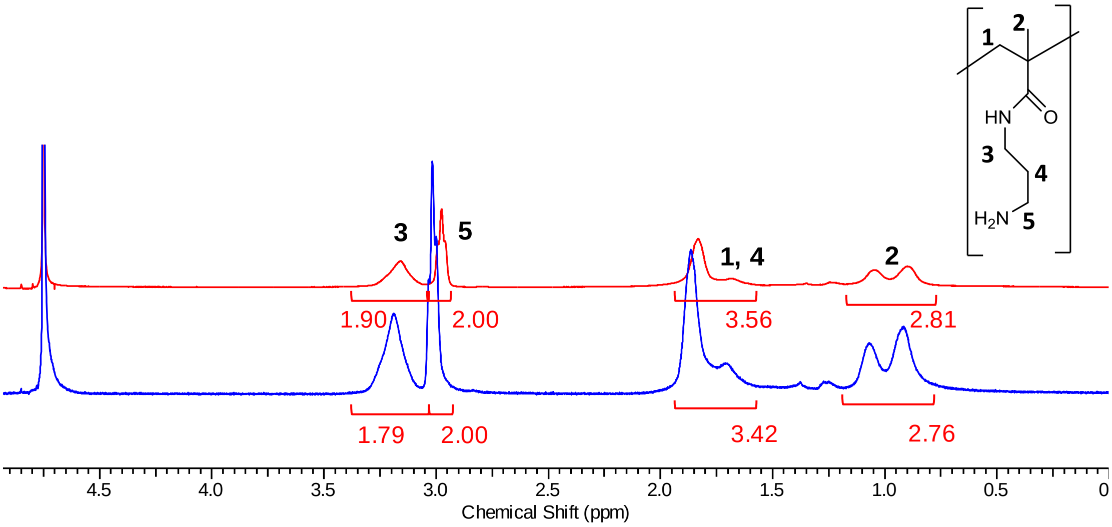

Figure S7. ^{1}H-NMR spectra of PAPMA homopolymer before (blue) and after (red) suspended to quaternization conditions as used for the copolymers systems.

Table S1. APMA content determined by ^{1}H-NMR (*signal not clearly expressed **signal is overlapped) and molecular weight determined by SEC (*** 0.45 μm instead of 0.2 μm PTFE filter, **** couldn't be filtered).

	Molar APMA content x_{ampa}	M_n [g/mol]	M_w [g/mol]	Đ
PMOTAC	---	33000	48000	1.5
P(MOTAC-*co*-APMA-5%)	3 %*	28000	37000	1.3
P(MOTAC-*co*-APMA-10%)	7 %*	37000	56000	1.5
P(MOTAC-*co*-APMA-15%)	17 %	33000	50000	1.5
P(MOTAC-*co*-APMA-30%)	31 %	40000***	116000***	2.9
POTAC	---	17000	19000	1.1
P(OTAC-*co*-APMA-5%)	10 %	18000	20000	1.1
P(OTAC-*co*-APMA-10%)	15 %	19000	21000	1.1
P(OTAC-*co*-APMA-15%)	20 %	18000	21000	1.2
P(OTAC-*co*-APMA-30%)	**	****	****	****
PMAPTAC	---	22000	26000	1.2
P(MAPTAC-*co*-APMA-5%)	5 %*	26000	32000	1.2
P(MAPTAC-*co*-APMA-10%)	15 %	24000	32000	1.3
P(MAPTAC-*co*-APMA-15%)	24 %	27000***	37000***	1.4
P(MAPTAC-*co*-APMA-30%)	35 %	****	****	****
PAPMA	100 %	52000	99000	1.9

Table S2. Degree of polymerization with respect to different monomer units present as derived from number averaged molecular weight using the APMA-content determined by ^{1}H-NMR.

	Overall degree of polymerization, DP_n	Number of APMA units
PMOTAC	160	-
P(MOTAC-*co*-APMA-5%)	140	4
P(MOTAC-*co*-APMA-10%)	180	12
P(MOTAC-*co*-APMA-15%)	160	28
P(MOTAC-*co*-APMA-30%)	200	62
POTAC	80	-
P(OTAC-*co*-APMA-5%)	94	9
P(OTAC-*co*-APMA-10%)	100	15
P(OTAC-*co*-APMA-15%)	95	19
P(OTAC-*co*-APMA-30%)	-	-
PMAPTAC	110	-
P(MAPTAC-*co*-APMA-5%)	120	6
P(MAPTAC-*co*-APMA-10%)	110	17
P(MAPTAC-*co*-APMA-15%)	130	31
P(MAPTAC-*co*-APMA-30%)	-	-
PAPMA	290	290

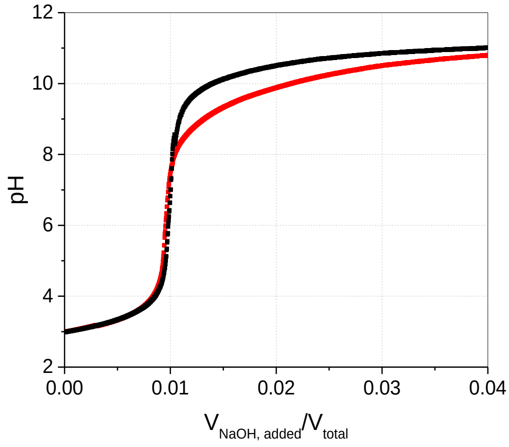

Figure S8. Potentiometric titration of HCl-containing solutions (+ 0.1 M KCl) either in absence of polymer (black, initial volume = 78.9 mL) or in presence of 10 mg of PAPMA (7×10^{-5} mol AMPA units; red; initial volume = 83.8 mL) with 0.1 M NaOH (added volume with respect to total volume, both solutions were acidified with 0.1 M HCl to pH 3 prior titration); results indicate that deprotonation of primary amine functions of PAPMA starts around pH 8.

Electrochemical characterization

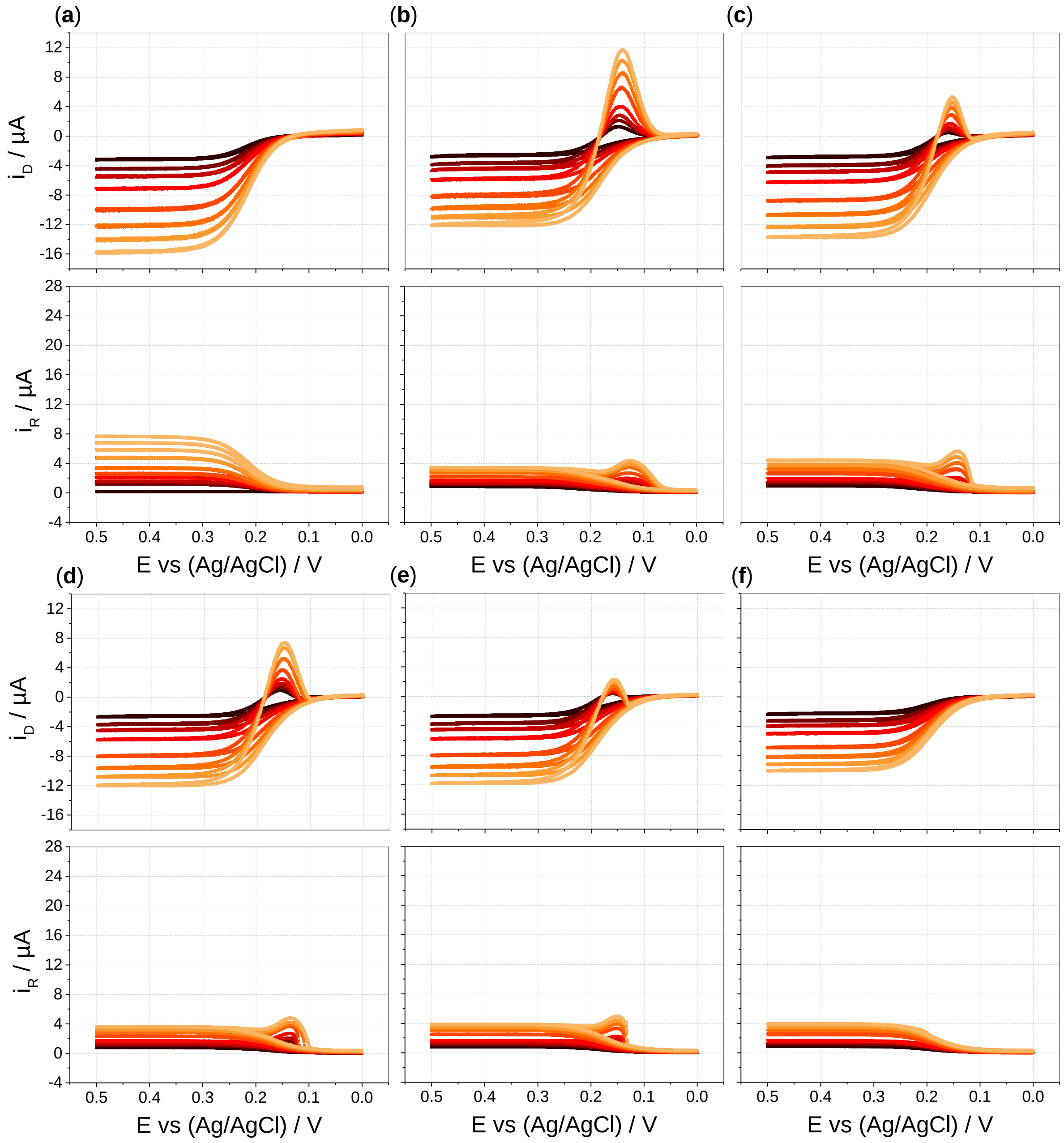

Figure S9. Hydrodynamic voltammograms of 0.13 mM HCF(II) in 0.1 M KCl in (**a**) absence and presence of 0.52 mM (with respect to quaternized units, *icr* = 1) (**b**) PMOTAC (**c**) P(MOTAC-*co*-APMA-5%), (**d**) P(MOTAC-*co*-APMA-10%), (**e**) P(MOTAC-*co*-APMA-15%), and (**f**) P(MOTAC-*co*-APMA-30%), (ring potential at 0 V vs. Ag/AgCl, disk potential scanned from 0 V to 0.5 V (forward scan) and back to 0 V (backward scan) vs. Ag/AgCl with ν = 5 mV/s, rotation rates increase as indicated from black to orange: 200, 400, 600, 1000, 2000, 4000, 5000 rpm).

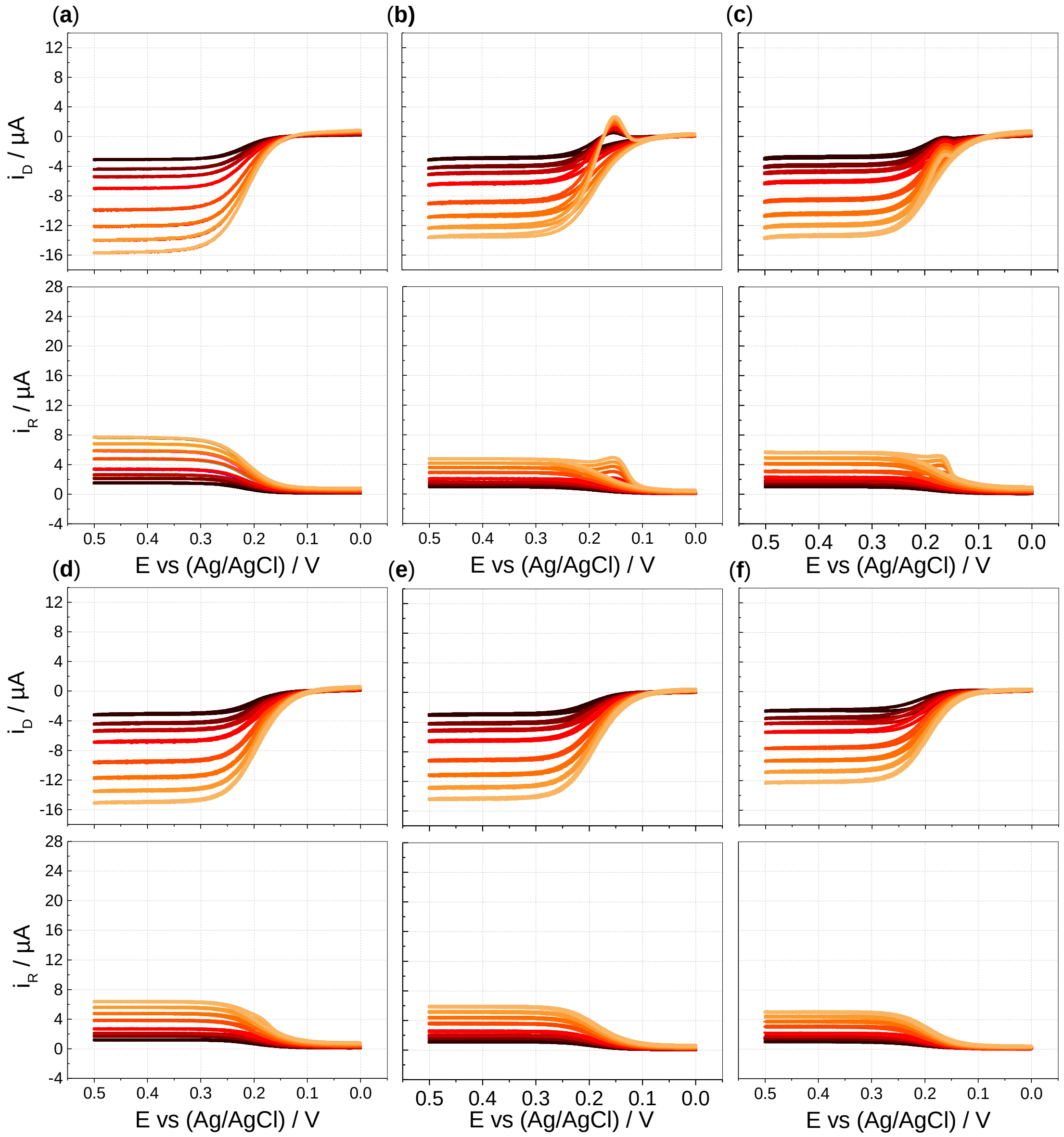

Figure S10. Hydrodynamic voltammograms of 0.13 mM HCF(II) in 0.1 M KCl in (**a**) absence and presence of 0.52 mM (with respect to quaternized units, *icr* = 1) (**b**) POTAC, (**c**) P(OTAC-*co*-APMA-5%), (**d**) P(OTAC-*co*-APMA-10%) (**e**) P(OTAC-*co*-APMA-15%) and (**f**) P(OTAC-*co*-APMA-30%) (ring potential at 0 V vs. Ag/AgCl, disk potential scanned from 0 V to 0.5 V (forward scan) and back to 0 V (backward scan) vs. Ag/AgCl with v = 5 mV/s, rotation rates increase as indicated from black to orange: 200, 400, 600, 1000, 2000, 4000, 5000 rpm).

The apparent diffusion coefficient of the HCF(II) in absence and presence of different polymers was determined via the Levich plot and equation (Figure S11). The kinematic viscosity was measured by means of an Ubbelohde tube immersed in a water bath at a temperature of 21 °C (using a PVS 1 LAUDA viscosity measuring system). The constant that correlates the kinematic viscosity with the measured time t for volume V to flow through the thin capillary is 0.004510 mm^2/s^2. Measurements of 0.1 M KCl, 0.13 mM HCF(II) in absence and presence of 0.52 mM PMOTAC yield a kinematic viscosity of 0.0101 +/− 0.002 cm^2/s. This value was used for all polymer systems assuming no change in the viscosity for the different polymers.

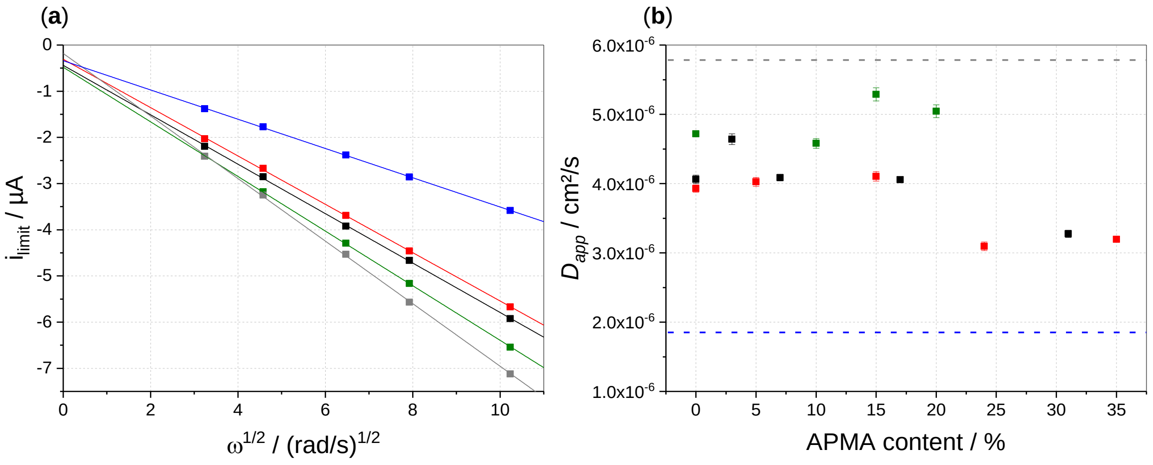

Figure S11. (**a**) Levich plots and (**b**) derived apparent diffusion coefficients vs. APMA molar content from hydrodynamic voltammograms of 0.13 mM HCF(II) in 0.1 M KCl in absence (grey) and presence of 0.52 mM PMOTAC (black), PMAPTAC (red), POTAC (green) and PAPMA (blue) (with respect to quaternized units/monomer units for PAPMA, *icr* = 1; dashed lines are limiting cases for HCF(II) diffusion in absence of polymer and in presence of PAPMA), rotation rates: 100–1000 rpm.

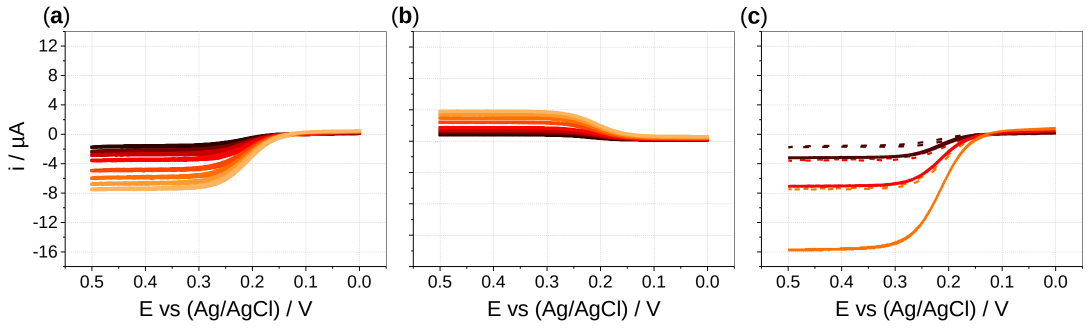

Figure S12. (**a**) Disk and (**b**) ring currents in hydrodynamic voltammograms of 0.13 mM HCF(II) in 0.1 M KCl in presence of 0.52 mM PAPMA (with respect to monomer units, *icr* = 1) (ring potential at 0 V vs. Ag/AgCl, disk potential scanned from 0 V to 0.5 V (forward scan) and back to 0 V (backward scan) vs. Ag/AgCl with *ν* = 5 mV/s, rotation rates increase from black to orange: 200, 400, 600, 1000, 2000, 4000, 5000 rpm) and (**c**) comparison of disk currents for 0.13 mM HCF(II) in 0.1 M KCl in absence (straight line) and presence of 0.52 mM PAPMA (dashed lines) at different rotation rates: 200 (black), 1000 (red), 5000 (orange).

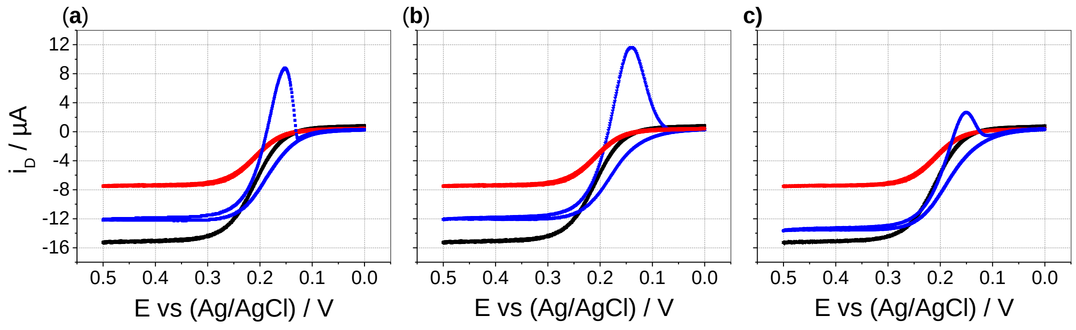

Figure S13. Disk currents in hydrodynamic voltammograms of 0.13 mM HCF(II) in 0.1 M KCl in absence (black) and presence of 0.52 mM PAPMA (with respect to monomer units, *icr* = 1) (red) and 0.52 mM of different polymers (with respect to quaternized polymer units): (**a**) PMAPTAC, (**b**) PMOTAC, (**c**) POTAC (disk potential scanned from 0 V to 0.5 V to 0 V vs. Ag/AgCl with ν = 5 mV/s).

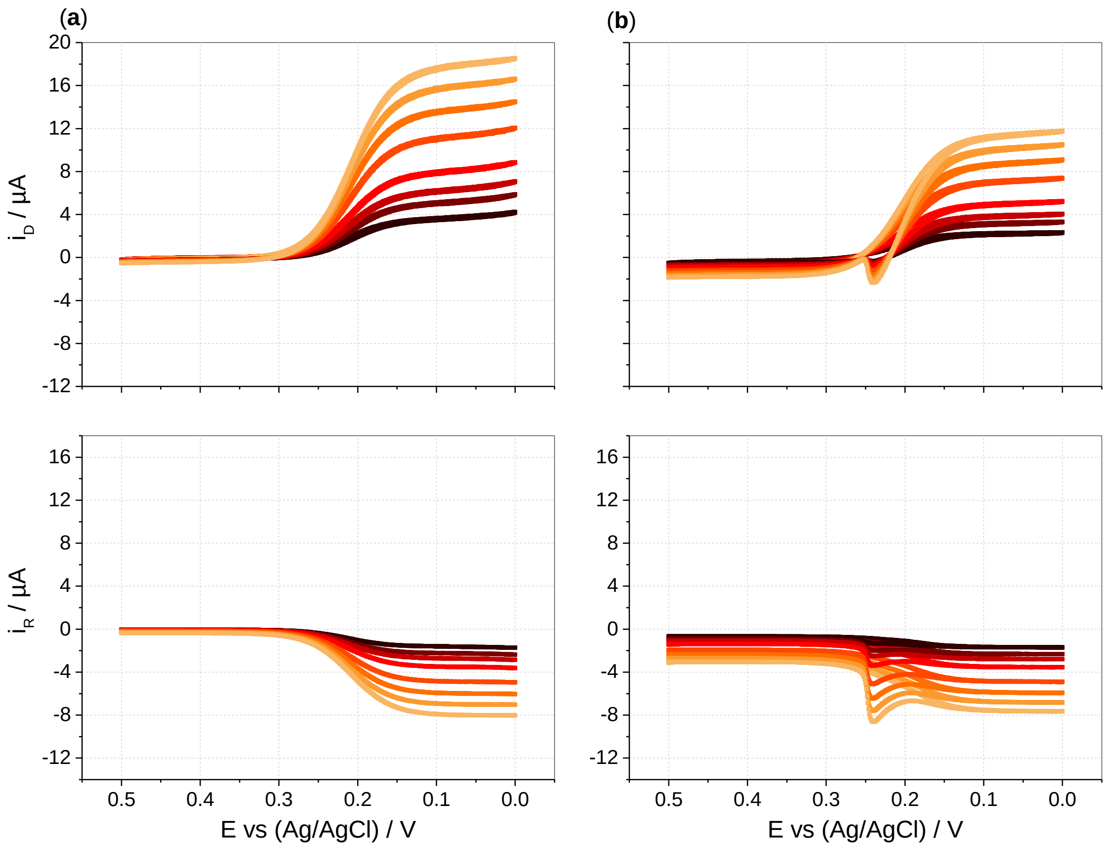

Figure S14. Hydrodynamic voltammograms of 0.13 mM HCF(III) in 0.1 KCl in absence (**a**) and presence (**b**) of 0.52 mM PAPMA (with respect to monomer units, *icr* = 1) (ring potential at 0.5 V vs. Ag/AgCl, disk potential scanned from 0.5 to 0 V (forward scan) and back to 0.5 V (backward scan) vs. Ag/AgCl with ν = 5 mV/s, rotation rates increase as indicated from black to orange: 200, 400, 600, 1000, 2000, 4000, 5000 rpm).

Collection efficiencies & deposition efficiencies

The so-called collection efficiency *N* is calculated to evaluate how much of the disk-generated ferricyanide is collected at the ring [1]. It is defined as the ratio of the limiting disk and ring current. For the HCF couple the collection efficiency is around 0.5. It is decreased upon film and complex formation.

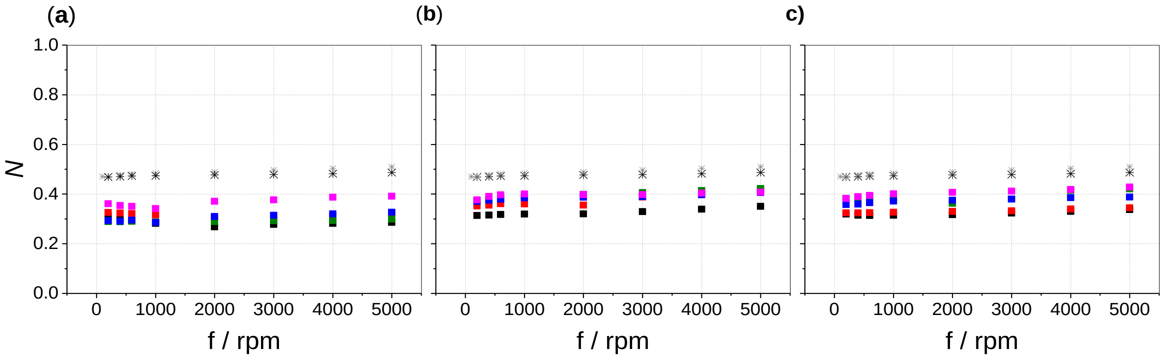

Figure S15. Collection efficiencies $N = i_{L,Ring}/i_{L,Disk}$ vs. rotation rate applied in hydrodynamic voltammograms of 0.13 mM HCF(II) in 0.1 M KCl in presence of 0.52 mM (with respect to quaternized polymer units, *icr* = 1) (**a**) P(MOTAC-*co*-APMA-x%), (**b**) P(OTAC-*co*-APMA-x%) and (**c**) P(MAPTAC-*co*-APMA-x%) with 0 % (black), 5 % (red), 10 % (green), 15 % (blue) and 30 % (magenta) percent of APMA, values for 0.13 mM HCF(II) in 0.1M KCl (black stars) in presence of 0.52 mM (with respect to monomeric units) of PAPMA (grey stars).

As the visible collection efficiencies *N* are the lowest for copolymers containing MOTAC units, this indicates that this polymer is the most effective with respect to film formation. Moreover, the increasing APMA-content results in increased *N* values for all polymers. This again shows that the film formation is reduced when decreasing the possibilities of bridging points. Collection efficiencies were determined from the average current (averaged for backward and forward scan) at 0.46 V.

The so-called deposition efficiencies were estimated corresponding to the given equation:

$$DE = \frac{\left(\int i_{disk,backward}\, dt - \int i_{disk,forward}\, dt\right) + \frac{\int i_{ring,backward}\, dt - \int i_{ring,forward}\, dt}{N}}{2\int i_{disk,forward}\, dt}$$

The charge required for the full reduction of the deposited ferricyanides is obtained by the difference of integration of the disk current (backward scan) and integration of the sigmoidal curve of ferrocyanide oxidation (forward scan). Thus, the cathodic disk peak current is determined. As some deposited ferricyanide is also detected at the ring, the corresponding difference in the integrals of backward and forward scan corrected by dividing by the *N* value is added to this expression. The calculated charge is compared to the total charge used for the oxidation of ferrocyanides. This is derived as twice the integral over the disk forward scan as the backward scan cannot be used due to the presence of the cathodic peak. Thus, the *DE* provides the information how much of the produced ferricyanide is used for film formation onto the disk electrode. Worthy to note, the baseline of forward and backward scan was compared and their difference subtracted from the cathodic disk peak area to eliminate contributions from capacitive background disk currents.

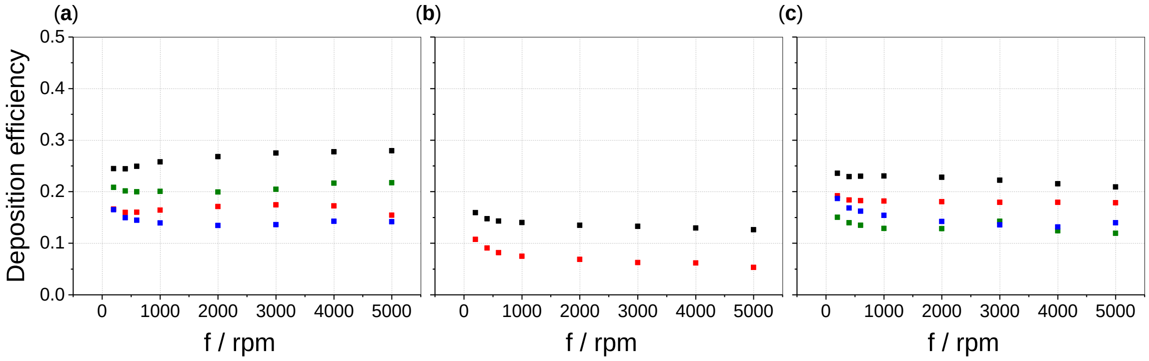

Figure S16. Deposition efficiencies *DE* vs. rotation rate *f* applied in hydrodynamic voltammograms of 0.13 mM HCF(II) in 0.1 M KCl in presence of 0.52 mM (with respect to quaternized polymer units, *icr* = 1) (**a**) P(MOTAC-*co*-APMA-x%), (**b**) P(OTAC-*co*-APMA-x%) and (**c**) P(MAPTAC-*co*-APMA-x%) with 0 % (black), 5 % (red), 10 % (green), 15 % (blue) and 30 % (magenta) percent of APMA. DE values were determined for measurements exhibiting a cathodic peak disk current.

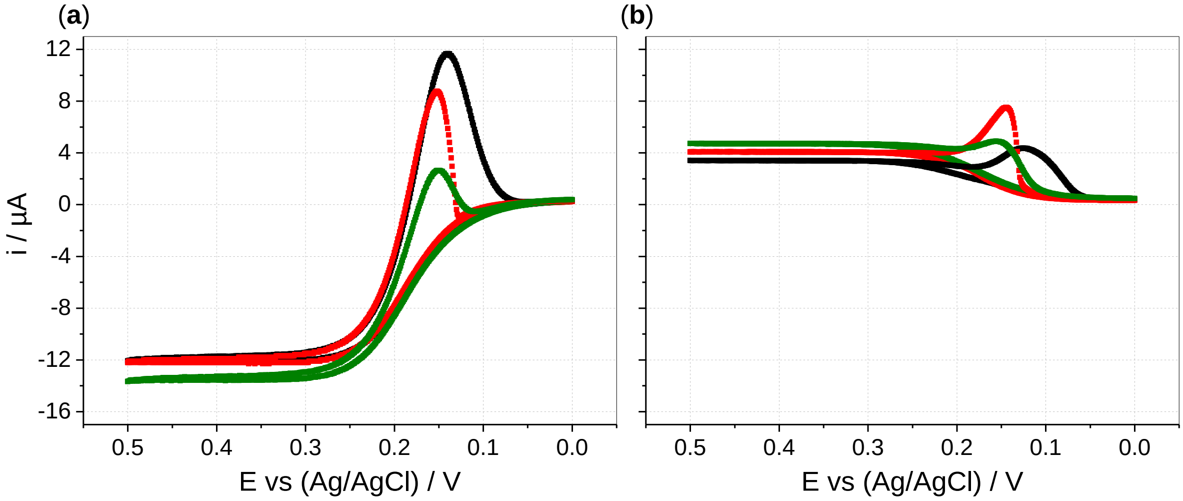

Figure S17. Disk (a) and ring (b) currents in hydrodynamic voltammograms at 5000 rpm of 0.13 mM HCF(II) in 0.1 M KCl in presence of 0.52 mM PMOTAC (black), PMAPTAC (red) and POTAC (green) (with respect to quaternized units, *icr* = 1) (ring potential at 0 V vs. Ag/AgCl, disk potential scanned from 0 V to 0.5 V (forward scan) and back to 0 V (backward scan) vs. Ag/AgCl with ν = 5 mV/s).

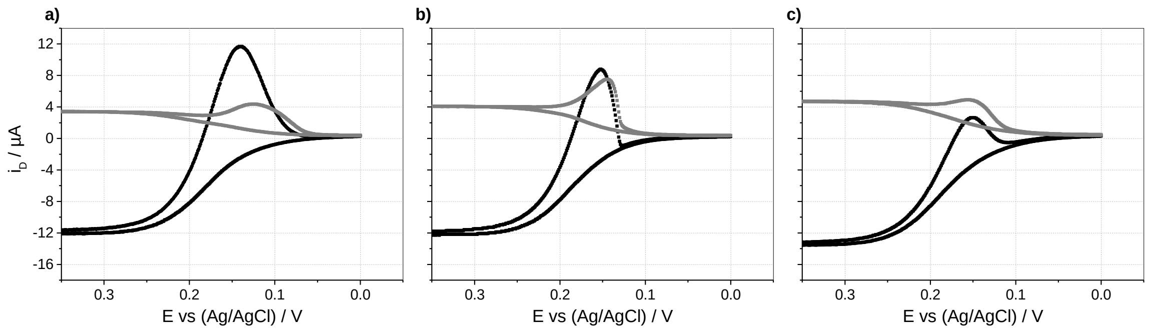

Figure S18. Zoom into the hydrodynamic voltammograms of 0.13 mM HCF(II) in 0.1 M KCl in presence of 0.52 mM (**a**) PMOTAC (**b**) PMAPTAC and (**c**) POTAC (with respect to quaternized units, *icr* = 1) with disk (black) and ring (grey) currents (ring potential at 0 V vs. Ag/AgCl, disk potential scanned from 0 V to 0.5 V (forward scan) and back to 0 V (backward scan) with v = 5 mV/s, rotation rate = 5000 rpm).

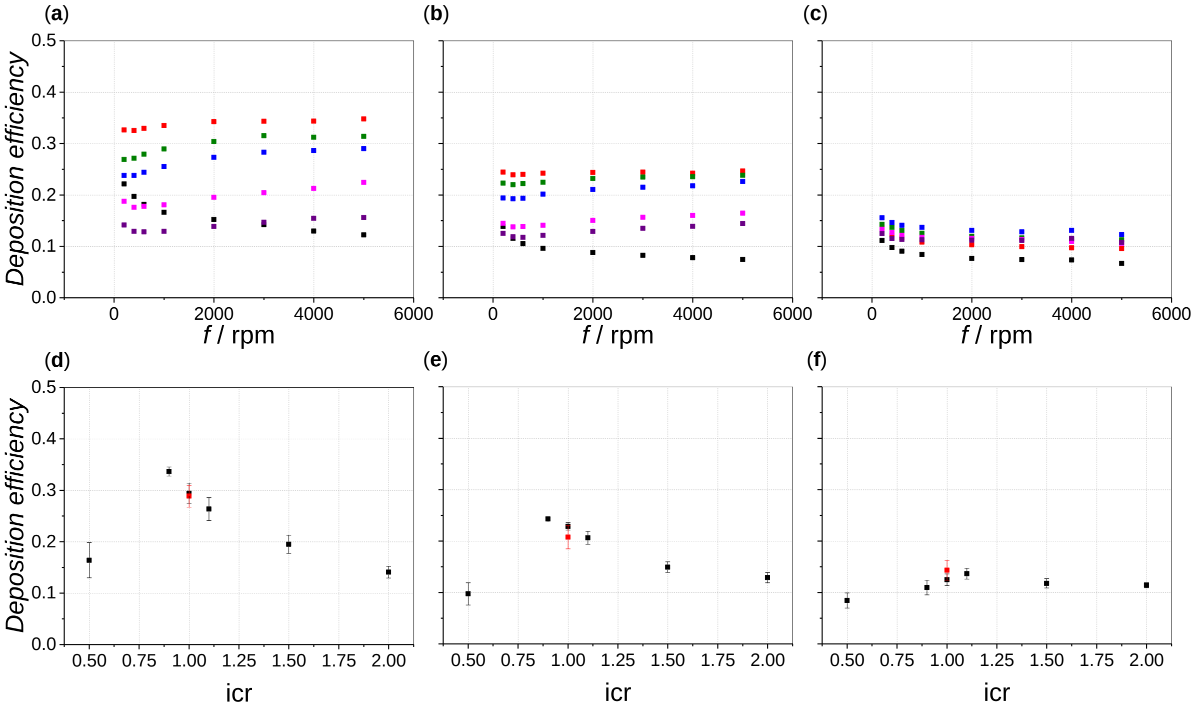

Figure S19: Deposition efficiencies *DE* vs. rotation rate *f* applied in hydrodynamic voltammograms of 0.13 mM HCF(II) in 0.1 M KCl in presence of 0.52 mM (witch respect to quaternized units) (**a**) PMOTAC, (**b**) PMAPTAC and (**c**) POTAC with *icr* values of 0.5 (black), 0.9 (red), 1 (green), 1.1 (blue), 1.5 (magenta) and 2 (purple) and deposition efficiencies averaged from 200–5000 rpm vs. *icr* of same measurements for (**d**) PMOTAC, (**e**) PMAPTAC and (**f**) POTAC (error bars indicate standard deviation from the mean, red values at *icr* 1 correspond to DE values in Figure 3 averaged from 200–5000 rpm).

Electrochemical Quartz Crystal Microbalance

Only minor changes in resistance during the measurements indicate a rigid film that allows for application of the Sauerbrey-Equation. This equation correlates changes in the eigenfrequency of a specifically cut quartz crystal to mass changes per area [2,3]. The so-called sensitivity factor C_f then only relies on fundamental properties of the Quartz crystal rendering any calibration to be redundant. The used AT-cut Quartz crystal exhibit a sensitivity factor of 56.6 Hz μg^{-1} cm^2.

$$\Delta f = -\left(\frac{2}{\rho_Q \vartheta_Q}\right) f^2 \Delta M = C_f \Delta M$$

ΔM: mass change per area Δf: change in frequency f: resonator frequency

ρ_Q: density of quartz plate ϑ_Q: shear wave velocity C_f: sensitivity factor

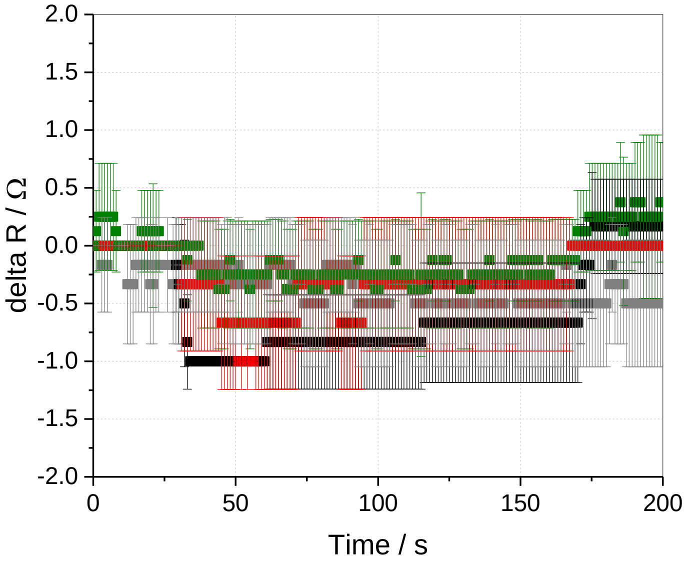

Figure S20. Change in resistance averaged over multiple measurements performed with an electrochemical quartz crystal microbalance during a voltage sweep (potential scanned from 0 to 0.5 to 0 V vs. Ag/AgCl with v = 5 mV/s) applied to solutions containing 0.1 M KCl, 0.13 mM HCF(II) in absence (grey) and presence of 0.52 mM (with respect to quaternized units, *icr* = 1) of PMOTAC (black), PMAPTAC (red) and POTAC (green).

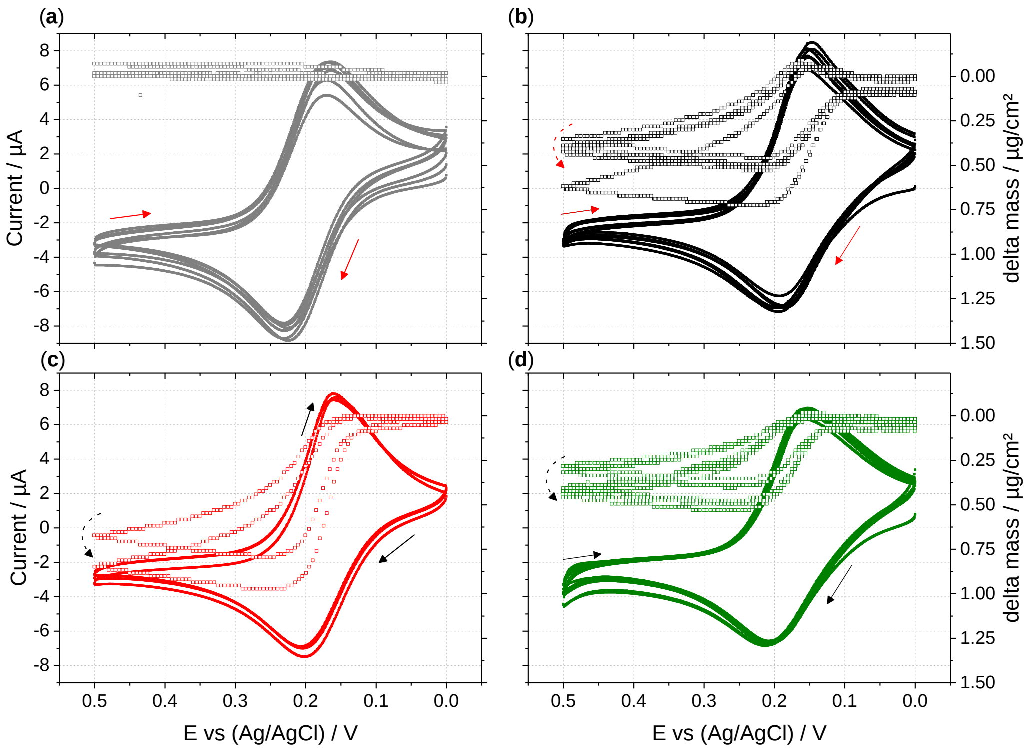

Figure S21. Electrochemical quartz crystal microbalance measurements during a voltage sweep (potential scanned from 0 to 0.5 to 0 V vs. Ag/AgCl with v = 5 mV/s) applied to 0.1M KCl, 0.13 mM HCF(II) in (**a**) absence and presence of 0.52 mM (**b**) PMOTAC, (**c**) PMAPTAC and (**d**) POTAC (with respect to quaternized units, *icr* = 1), closed symbols refer to measured cyclic voltammograms (left vertical axis) and open symbols refer to changes in delta mass (right vertical axis) vs. voltage.

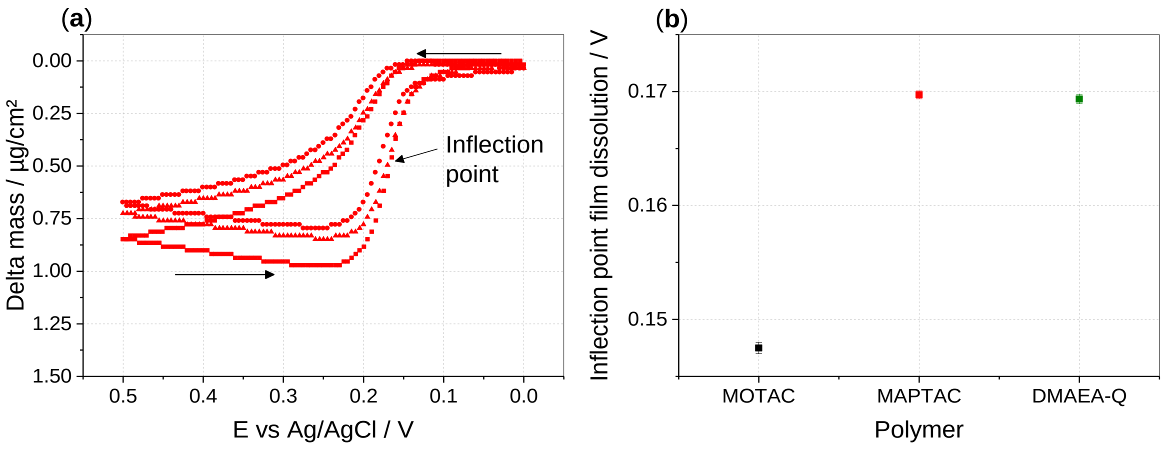

Figure S22. (**a**) Change in delta mass during cyclic voltammetry (potential scanned from 0 to 0.5 to 0 V vs. Ag/AgCl with v = 5 mV/s) of 0.13 mM HCF(II) in 0.1 M KCl in presence of 0.52 mM PMAPTAC (with respect to quaternized units, *icr* = 1), arrows mark direction and the inflection point used for (**b**) potential inflection point during film dissolution determined by fitting a sigmoidal Boltzmann function.

References

1. Bard, A.J.,Faulkner, L.R., *Electrochemical Methods: Fundamentals and Applications*. 2nd ed.; Wiley: New York, United States of America; 2000; pp. 348–360, 9780471043720.
2. Sauerbrey, G., Verwendung von Schwingquarzen zur Wägung dünner Schichten und zur Mikrowägung. *Z. Phys.* **1959**, *155*, 206–222, 10.1007/bf01337937.
3. Hillman, A.R., The Electrochemical Quartz Crystal Microbalance. In *Encyclopedia of Electrochemistry*; Wiley-VCH Verlag GmbH & Co. KGaA, 2007; pp. 230–289, 9783527610426.
